# Enhanced activity of pyramidal neurons in the infralimbic cortex drives anxiety behavior

**DOI:** 10.1371/journal.pone.0210949

**Published:** 2019-01-24

**Authors:** Laura Berg, Josephine Eckardt, Olivia Andrea Masseck

**Affiliations:** 1 Advanced Fluorescence Microscopy, Ruhr University Bochum, Bochum, Germany; 2 Department of Systems Neuroscience Ruhr University Bochum, Bochum, Germany; 3 University of Bremen, Synthetic Biology, Bremen, Germany; Technion Israel Institute of Technology, ISRAEL

## Abstract

We show that in an animal model of anxiety the overall excitation, particularly in the infralimbic region of the medial prefrontal cortex (IL), is increased and that the activity ratio between excitatory pyramidal neurons and inhibitory interneurons (AR PN/IN) is shifted towards excitation. The same change in AR PN/IN is evident for wildtype mice, which have been exposed to an anxiety stimulus. We hypothesize, that an elevated activity and the imbalance of excitation (PN) and inhibition (IN) within the neuronal microcircuitry of the prefrontal cortex is responsible for anxiety behaviour and employed optogenetic methods in freely moving mice to verify our findings. Consistent with our hypothesis elevation of pyramidal neuron activity in the infralimbic region of the prefrontal cortex significantly enhanced anxiety levels in several behavioural tasks by shifting the AR PN/IN to excitation, without affecting motor behaviour, thus revealing a novel mechanism by which anxiety is facilitated.

## Introduction

Anxiety disorders belong to the most common mental illnesses within the western world affecting a total amount of about 20% of the human population, a lifetime prevalence of nearly 30% and with a steadily increasing occurrence [1–3]. One of the key areas involved in the expression of anxiety and fear, beside the hippocampus and the amygdala, is the infalimbic region (IL) of the medial prefrontal cortex (mPFC) [4–9], which is the rodent homolog to the anterior cingulate cortex of humans. In contrast to fear, which is a specific in time limited response to a threatening stimulus, often investigated with fear conditioning, anxiety is a sustained negative emotional state, in which unknown or uncertain situations are encountered with defensive behaviour [10].

Studies have repeatedly shown, that neuronal excitability is increased in the prefrontal cortex of patients with anxiety disorders and in animal models of anxiety [9,11,12]. Consequently, mainly disturbances in GABAergic neurotransmission have been associated with the manifestation and appearance of anxiety disorders [4,13]. Treatments of choice for generalized anxiety disorders are in many cases benzodiazepine, which act on GABAergic receptors and enhance inhibitory neurotransmission in general [14]. But also contradictory result have been obtained, lesions of the mPFC, or inhibition did not affect anxiety behaviour at all or elicit even opposing effects, i.e. anxiogenic or anxiolysis, on innate fear and learned fear, pointing to a highly complex role of the mPFC in anxiety [15,16].

One striking hypothesis for neuronal mechanism underlying various psychiatric diseases, is that changes in cortical excitation inhibition (E/I) balance are responsible for observed cognitive deficits [3].

Neuromodulatory systems, such as the noradrenergic or in particular the serotonergic system seem to contribute to the development and manifestation of anxiety [17,18]. For example, anxiety disorders such as panic, posttraumatic stress, social phobia or obsessive-compulsive disorder are commonly treated by selective serotonin reuptake inhibitors (SSRIs) suggesting an important role of 5-HT in these diseases [19]. As the serotonergic system projects to all parts of the well known tripartite anxiety circuitry, namely the ventral Hippocampus, the Amygdala and the prefrontal cortex [4,20–22]it is likely that the serotonergic system might be potent modulator of anxiety behaviour. The mPFC receives massive input from the serotonergic system and 5-HT affects postsynaptic excitatory, as well as inhibitory neurons mainly by 5-HT_1A_ receptors [8,17]. Low levels of 5-HT_1A_ receptor have been associated with the manifestation and development of anxiety disorders [18] and 5-HT_1A_ knock mice are a well established genetic model of anxiety [23–25]. A previous study indicates that 5-HT_1A_ heteroreceptor expression in cortex and hippocampus is primarily responsible for the anxiety phenotype [26]. In conjunction, 5-HT_1A_ knock out mice show abnormalities within the GABA-glutamate system of the prefrontal cortex [27] pointing again to an imbalance of excitatory and inhibitory neurotransmission within the prefrontal cortex, probably mediated by disturbances in the 5-HT system. However, so far the underlying neuronal mechanisms are still largely unknown and despite numerous studies on 5-HT signalling and anxiety, their involvement in modulating the ratio between excitatory pyramidal and inhibitory interneurons activity (AR PN/IN) has not been scrutinized.

## Material and methods

All experiments in this study were approved by the Institutional Animal Research Facility (Landesamt für Natur, Umwelt und Verbraucherschutz NRW). Mice were housed in standardized cages and maintained on a 12:12 (h) light-dark cycle (lights on from10.00 pm-10.00am) in a temperature and humidity controlled scantainer. Food and water were available ad libitum. All behavioral experiments were performed in the main activity phase of the animals. Experiments were performed in C56Bl/6J mice (Jackson Laboratory) and in two different transgenic mousselines with a C56Bl/6J background obtained from collaborators. For characterizing the activity pattern of different neuron types during anxiety 5-HT_1A_(-/-) mice (kindly provided by L. Tecott, University of California San Fransisco)[23] were used. To specifically express ChR2 or a fluorescent control plasmid (td-tomato) in pyramidal neurons Nex-Cre(-/-) mice were used (kindly provided by K. Nave and S. Goebbels, Max Planck Institute für Experimentelle Medizin) [28].

### Viral vectors and surgery

3–6 month old C56Bl/6J mice were stereotaxically injected with Fluorogold (Thermo Fisher, H22845) for retrograde tracing into the dorsal raphe nucleus (AP -4.5 mm, ML 0 mm, DV -2.5mm, -2mm, -1.5mm), the IL region (AP + 1.66 mm, ML 0.3 mm left, DV -2mm, -1.8mm, -1.6mm) or the amygdala (AP -1.58 mm, ML -3 mm left, DV -3.8mm). Nex-Cre(-/-) mice were injected with AAVs, encoding for Cre depended expression to target ChR2 (AAV9.EF1a.DIO.hChR2(H134R)-eYFP.WPRE.hGH (Adgene 20298, Penn Vector Core)) or td-tomato (pAAV1.CAG.FLEX.tdTomato.WPRE.bGH (Penn Vector Core, Adgene 51503)) to pyramidal neurons in the IL region.

All surgical procedures were performed under anesthesia with Isofluran (Initial 5%, 2% for surgery). Pressure injections of virus or tracer, by customized glass pipettes (tip diameter 10μm), were performed at various depths to ensure proper virus spreading within the target area. For optogenetic experiments a customized ceramic ferrule (Thorlabs, 1,25mm diameter with 230μm bore hole, CFLC230-10) with an opitical fiber (Thorlabs, FT200EMT, length 1.8mm, diameter 200μm) was implanted directly above the IL region for optical stimulation. Therefor the skull was prepared with the primer and adhesive of Optibond FL (Kerr) and the implant was fixed with Gradia direkt—Flo dental cement (Henry Schein, 103322) and cured with UV light. After surgery animals received subcortical injection of Carprofen (2 mg/kg) for analgesia and 0.1ml glucose. Animals were from now on housed individually and were allowed to recover at least for 14 days before behavioral experiments were performed. To identify projections between the prefrontal cortex and the serotonergic system and vice versa, as also between the prefrontal cortex and the amygdala, we injected Fluorogold dissolved in NaCl into the dorsal raphe, the IL region or the amygdala respectively. Mice were perfused after 5–7 days of retrograde transport and injection sites were verified by fluorescence microscopy and immunhistochemistry.

### Immunohistochemistry

For immunohistochemical analysis mice were perfused transcardial after exposure to different conditions: 5-HT_1A_(-/-) mice and Wt littermates were either sacrificed naïve, i.e. directly from their homecages, or 90 minutes after a 5 minute exposure to the Elevated-Plus maze (anxiety stimulus). Nex-Cre(-/-) mice were either sacrificed naïve, 90 after 5 minutes exposure to the Open-Field, or 90 minutes after 5 minutes within the Open-Field plus light stimulation (473 nm, 20Hz) [29,30].

Mice were euthanized with an overdose of Ketamin (0,33mg/g) i.p. and brains were post fixed in 4% PFA in PBS overnight and processed in 30% sucrose for cryosectioning. Obtained brain slices (40μm) were washed 3x times in PBS. After blocking in 3% donkey serum in 0.3% PBST slices were stained overnight in 1,5% donkey serum in 0.3% PBST at room temperature with rabbit-anti-c-Fos (226003, Synaptic Systems), mouse-anti-Gad67 (MAB5406, Merck Millipore) and sheep-anti-TPH (MBS502127, MyBioSoure) for the DRN and rabbit-anti-c-Fos (226003, Synaptic Systems, 1:1000), mouse-anti-Gad67 (MAB5406, Merck Millipore, 1:500) and goat-anti-CamKIIa (sc-5391, Santa Cruz Biotechnology, 1:300) for the IL region and amygdala.

The next day, after 3 additional washing steps in PBS, slices were incubated, for 1 h at room temperature, with the secondary antibody. For the 5-HT_1A_(-/-) mice Cye3-donkey-anti-rabbit (711-165-152, Jackson ImmunoResearch, 1:500), Alexa488-donkey-anti-sheep (A-11015, Thermo Fisher, 1:500) and DyLight647-donkey-anti-mouse (715-605-150, Jackson ImmunoResearch, 1:500) for the DRN and Cye3-donkey-anti-rabbit (711-165-152, Jackson ImmunoResearch, 1:500), Alexa488-donkey-anti-goat (A-11055, Thermo Fisher, 1:500) and DyLight647-donkey-anti-mouse (715-605-150, Jackson ImmunoResearch, 1:500) for the IL region. For the Nex-Cre(-/-)-ChR2-eYFP mice it was DyLight405-donkey-anti-sheep (DkxSh-003-F405NHSX, ImmunoReagents, 1:500), DyLight550-donkey-anti-rabbit (SA5-10039, Thermo Fisher, 1:500) and Alexa647-donkey-anti-mouse (715-605-150, Jackson ImmunoResearch, 1:500) for the DRN and DyLight405-donkey-anti-goat (705-475-003, Jackson ImmunoResearch, 1:500), DyLight550-donkey-anti-rabbit (SA5-10039, Thermo Fisher, 1:500) and Alexa647-donkey-anti-mouse (715-605-150, Jackson ImmunoResearch, 1:500) for the IL region and the amygdala. For the Nex-Cre(-/-)-dtomato mice it was DyLight405-Donkey-anti-sheep (DkxSh-003-F405NHSX, ImmunoReagents, 1:500), DyLight488-donkey-anti-mouse (DkxMu-003-D488NHSX, ImmunoReagents, 1:500) and Alexa647-donkey-anti-rabbit (711-605-152, Jackson ImmunoResearch, 1:500) for the DRN and DyLight405-donkey-anti-goat (705-475-003, Jackson ImmunoResearch, 1:500), DyLight488-donkey-anti-mouse (DkxMu-003-D488NHSX, ImmunoReagents, 1:500) and Alexa647-donkey-anti-rabbit (711-605-152, Jackson ImmunoResearch, 1:500) for the IL region and the amygdala.

Slices were mounted on Superfrost Plus microscope slides (J1800AMNZ, Thermo Scientific) with Roti-Mount FluorCare (HP19.1, Carl Roth) and Cover slips (24x60 mm #1, Menzel-Gläser) and analyzed with a laser scanning confocal microscope (SP5, or SP8, Leica).

Brian slices from fluorogold injected brains were either directly mounted on Superfrost Plus microscope slides (J1800AMNZ, Thermo Scientific) with Roti-Mount FluorCare containing DAPI (HP19.1, Carl Roth) and Cover slips (24x60 mm #1, Menzel-Gläser), or stained against TPH in the DRN or CamKII in amygdala and IL with the same antibodies as for the Nex-Cre mice.

### Behavioral experiments

We used male mice for all behavioral experiments. Before implantation mice were housed in groups. Each mouse was tested only once in each behavioral test and at least one week was in between different anxiety tests. Trials between groups were always interleaved.

#### Elevated-Plus maze

For the Elevated-Plus Maze 5-HT_1A_(-/-) mice were transported individually from the mouse facility to the experimental room. Prior to the experiment mice were marked with a green label to be later recognized by the tracking software (TDT). The Elevated-Plus maze consists of two open (total length 33,5cm, 5cm wide) and two closed arms (total length 33,5cm with a 17cm high wall, 5cm wide) all connected to a center platform in the middle. After 10 minutes of habituation to the experimental room, mice were placed at the center of the maze facing the open arm directed to the experimenter. Its behavior was recorded for 5 minutes and analyzed by a customized Matlab routine. For optogenetic stimulation of pyramidal neurons in the cortex of Nex-Cre(-/-) mice, optogenetic implants were connected via a sleeve and optical cable to the light source (473 nm LED, Pizmatix, Optogenetics-LED-STSI). After habituation in an extra waiting cage (10 min) mice were placed in the center of the EPM, directly facing the experimenter. The behavior was recorded and analyzed by Ethovision XT (Noldus) for 18 minutes with alternating 3-minutes off and on trials [31]. In three trials (1,3,5) no light stimulation occurred, whereas in all remaining trials (2,4,6) light stimulation was on. Videos were analyzed by the tracking software EthoVision XT. Time mice spend on the open arms, open arm entries and distance moved in total was quantified.

#### Open-Field

For the openfield test mice were transported individually from the mouse facility to the experimental room. Optogenetic implants were connected via a sleeve and optical cable to the stimulating light source (473 nm LED, Prizmatix, Optogenetics-LED-STSI). After connecting animals to the light stimulation fiber, mice were allowed to habituate in an extra waiting cage, which was positioned outside of the testing arena.

The Open-Field arena is a squared plexiglasbox with sandblasted walls (50x50x50cm). After 10 minutes of habituation mice were placed into the left lower corner, facing towards the center. In total, mice behavior was recorded for 20min. Obtained data was analyzed offline by EthoVision XT (Noldus). Each experiment consisted of four 5-minute trials with alternating light on and light off [31]. In two trials (2,4) light stimulation was switched on (20Hz, 1mW) [29,30], in the remaining trials (1,3) no stimulation was done. The arena was divided into 16 equal squares, whereby the 4 inner squares build the center (25x25cm). Videos were analyzed automatically with a tracking software (Noldus) for time spend in the center of the open field, entries to the center and distance traveled.

#### Novelty-Suppressed feeding

For the Novelty-Suppressed Feeding test mice were deprived from food for 24h but had access to water ad libitum. On the testing day mice were transported individually from the facility to the experimental room. Optogenetic implants were connected via a sleeve and optical cable to the stimulating light source (473 nm LED, Prizmatix, Optogenetics-LED-STSI). After connecting to the light stimulation fiber, mice were allowed to habituate in an extra waiting cage, which was positioned outside of the testing arena. After 10 minutes of habituation mice were placed in the left lower corner of the Open field arena (see openfield test). The floor of the openfield was covered with litter and a standard food pellet, situated on a 5x5 cm filter paper, was placed in the middle of the center. Recordings were stopped as soon as mice started feeding, defined as mice holding the food pellet in their fore pads and biting into it. During the experiment mice were either stimulated by light (20Hz, 1,mW) (Liu et al. 2014, Zhao et al., 2011), or only connected to the optical fiber without performing light stimulation. Subsequently, mice were allowed to eat their food pellet for additional 5 minutes in their home cage. Afterwards the food pellet was weighted to calculate the amount of food intake. For analysis time to feed, and time in the center of the openfield during the first minute were taken into consideration.

#### Data analysis

Data were analyzed by EthoVision XT (Noldus), ImageJ, SigmaPlot, SigmaStat. All obtained images were processed in ImageJ for contrast and brightness. Overlays of c-Fos with either Gad67 or CamKIIa/TPH were created and all cells of the specific types were counted in ImageJ, which expressed c-Fos, as also all c-Fos positive cells in total.

Heatmaps were produced in EthoVision XT, where color codes represent time spent in each location. Data extraction and collection was done automatically from recorded videos by EthoVision XT. Data were then further analyzed in SigmaPlot and tested for normality.

## Results

### C-fos expression in the dorsal raphe nucleus during anxiety

**Fig 1:**
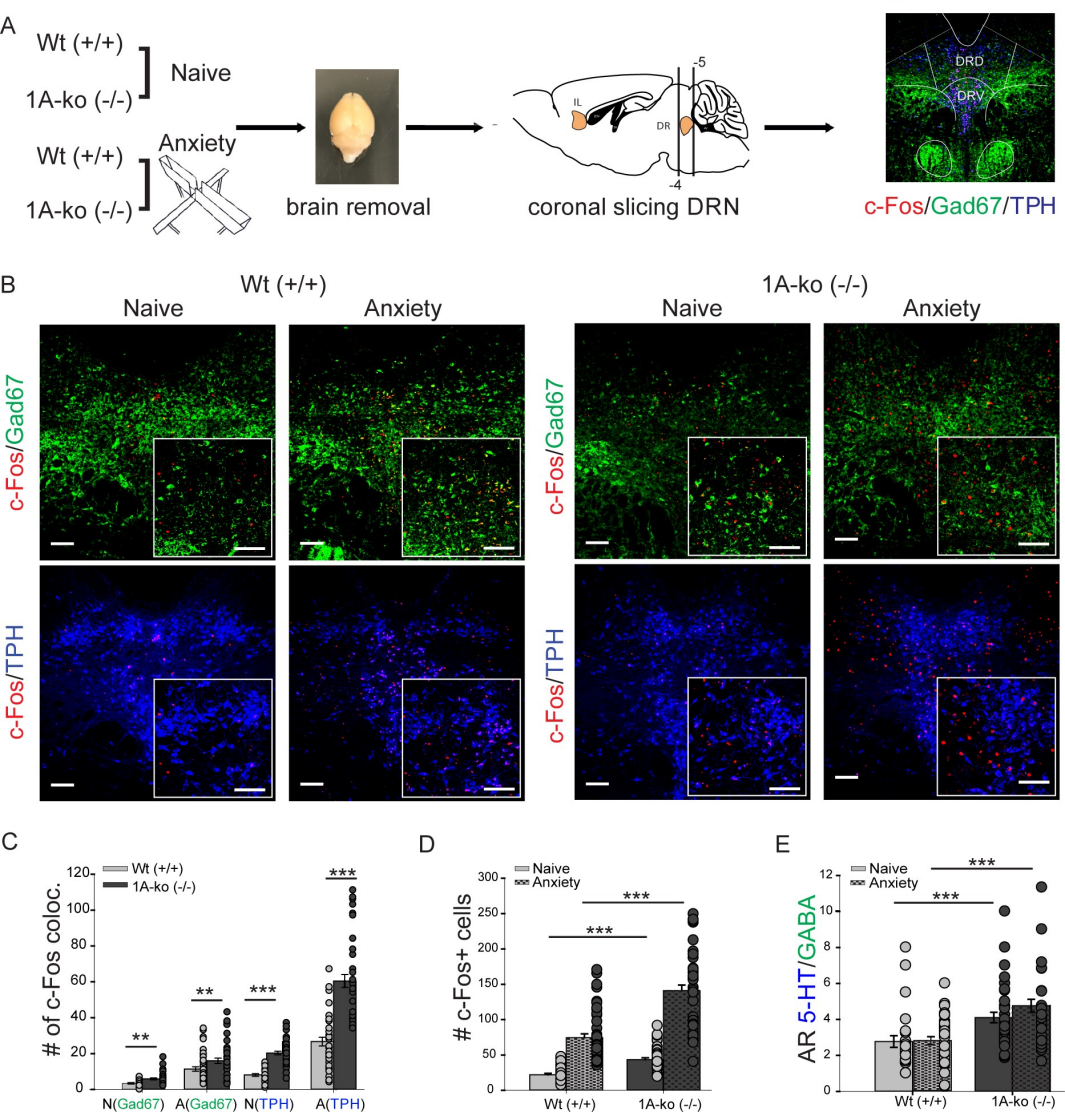
AR 5-HT/GABA in the serotonergic system is not changed due to anxiety. **(A)** experimental workflow of c-fos stainings. DRN dorsal raphe nucleus, c-fos marked in red, Gad67 fluorescence labeling of GABAergic neurons (green), TPH fluorescence labeling of serotonergic neurons (red). **(B)** confocal images showing c-fos expression in wildtype (Wt) and 5-HT_1A_(-/-) knockout mice (1A-ko). C-fos in red. Insets show high magnification. Scale bar 150μm, insets 100 μm. **(C)** colocalization of c-fos expression in GABAergic and serotonergic cells (analysis of 42 brain slices from 3 brains per group). N naive, A anxiety. N(Gad67) Wt 3.34±0.37, 1A-ko 5.88±0.53, Mann Whitney Rank Sum test, p = 0.003, A(Gad67) Wt 11.4±1.25, 1A-ko 15.98±1.52, Mann Whitney Rank Sum, p = 0.01, N(TPH) Wt 8.19±0.81, 1A-ko 20.29±1, two tailed t-test t = -8.595, p≤0.001, A(TPH) Wt 26.69±2.38 1A-ko 60.48±3.58, Mann Whitney Rank Sum test, p≤0.001. **(D)** total amount of c-fos expressing cells. Wt naïve 22.14±2.06, 1A-ko naïve 43.43±2.47, two-tailed t-test t = -5.978, p≤0.001, Wt anxiety 74.47±5.48, 1A-ko anxiety 141.14±7.8 Mann Whitney Rank Sum test p≤0.001 **(E)** activity ratio between serotonergic neurons and interneurons (AR 5-HT/GABA) calculated as ratio between c-fos positive serotonergic and c-fos positive GABAergic neurons. Wt naïve 2.77±0.33, Wt anxiety 2.79±0.19, Mann Whitney Rank Sum test, p = 0.372, 1A-ko naïve 4.11±0.27, 1A-ko anxiety 4.76±0.36, Mann Whitney Rank Sum test, p = 0.238. Values are mean ± S.E.M. ** indicate significant differences (p≤ 0.01), ***indicate significant differences (p≤ 0.001).

To test now whether disturbed serotonergic neurotransmission might contribute to changes in cortical activity patterns we performed immunohistochemical analyses of neuronal activity in 5-HT_1A_(-/-) knockout mice (Fig 1), which exhibit enhanced anxiety levels in comparison to wildtype mice (S1 Fig). We examined anxiety behaviour in two behavioural tests, the Elevated Plus Maze (EPM) and the Novelty Suppressed Feeding test (NSF), both tests revealed enhanced anxiety in 5-HT_1A_(-/-) knockout mice. In the EPM time spent in open arms (Wt 47.91±6.12s; 1A-ko 31.59±4.34s, p = 0.036) and open arm entries (Wt 10.6±1.25; 1A-ko 7.28±0.66, p = 0.015) were significantly decreased (S1B and S1C Fig), while the overall distance moved (Wt 3859.34±979.26cm, 1A-ko 4373±669.08cm, p = 0.487) was not affected at all (S1D Fig). Just like in the EPM also the NSF confirmed enhanced anxiety in 5-HT_1A_(-/-) knockout mice, time spent in the center of the arena (Wt = 27.32±3.81s, 1A-ko = 17.57±2.6s, p = 0.042) is significantly decreased and time till feeding showed a trend towards longer waiting times (Wt 118.56±19.16s, 1A-ko 146.69±36.67s, p = 0.772) (S1F and S1G Fig).

Having confirmed increased anxiety in 5-HT_1A_(-/-) we started to compare neuronal activity, i.e. c-fos expression, in wildtype and 5-HT_1A_(-/-) knockout mice in naïve (homecage) and fearful situations (Elevated-Plus Maze) (Fig 1A). For this, animals were subjected to either a neutral situation, i.e. remaining in their homecage (naïve) or underwent an anxiety provoking Elevated-Plus Maze test for 5 min followed by additional 90 min in their homecage, in which c-fos expression reaches its maximum. Brains were removed and coronal sections of the DRN and IL were immunolabelled for the neuronal activity marker c-fos (Fig 1A). In the literature extensive interconnectivity between the serotonergic system, mainly the dorsal raphe nucleus (DRN) and the prefrontal cortex is described [32,33]. To verify this interconnectivity we decided to perform tracer injections in the IL and DRN (S2 Fig). Injections of fluorogold in the IL revealed retrogradly stained neurons in the DRN (S2A Fig) and specific expression of td-tomato in pyramidal neurons of NEX-Cre revealed anterogradly labelled fiber terminals in the DRN (S2B Fig). Vice versa, fluorogold injections in the DRN labelled pyramidal neurons in the IL region (S2C Fig).

Once we have revisited extensive connections between the DRN and the IL, we focused our analysis of the activity marker c-fos in naïve and fearful situations first within the DRN.

Our analysis revealed an increase of total c-fos expression in TPH and GABAergic neurons in anxiety provoking situations in Wt as well as in 5-HT_1A_(-/-) mice (Fig 1B, 1C and 1D). Whereby in all conditions and cell types the number of c-fos is increased in 5-HT_1A_(-/-) mice (Wt naïve 22.14±2.06, 1A-ko naïve 43.43±2.47, p≤0.001, Wt anxiety 74.47±5.48, 1A-ko anxiety 141.14±7.8 p≤0.001) (Fig 1C and 1D), probably due to missing feedback inhibition of the 5-HT_1A_ receptor [34]. However, ratios between c-fos expressing serotonergic (TPH) and c-fos expressing GABAergic neurons is not changed due to anxiety in Wt (Wt naïve 2.77±0.33, Wt anxiety 2.79±0.19, p = 0.372) as well as in 5-HT_1A_(-/-) mice (1A-ko naïve 4.11±0.27, 1A-ko 24.76±0.36, p = 0.238) (Fig 1E). Hence no significant changes in AR 5-HT/GABA between naïve and anxiety condition is present in the DRN (Fig 1E).

### C-Fos expression in the infralimbic cortex in anxiety

**Fig 2:**
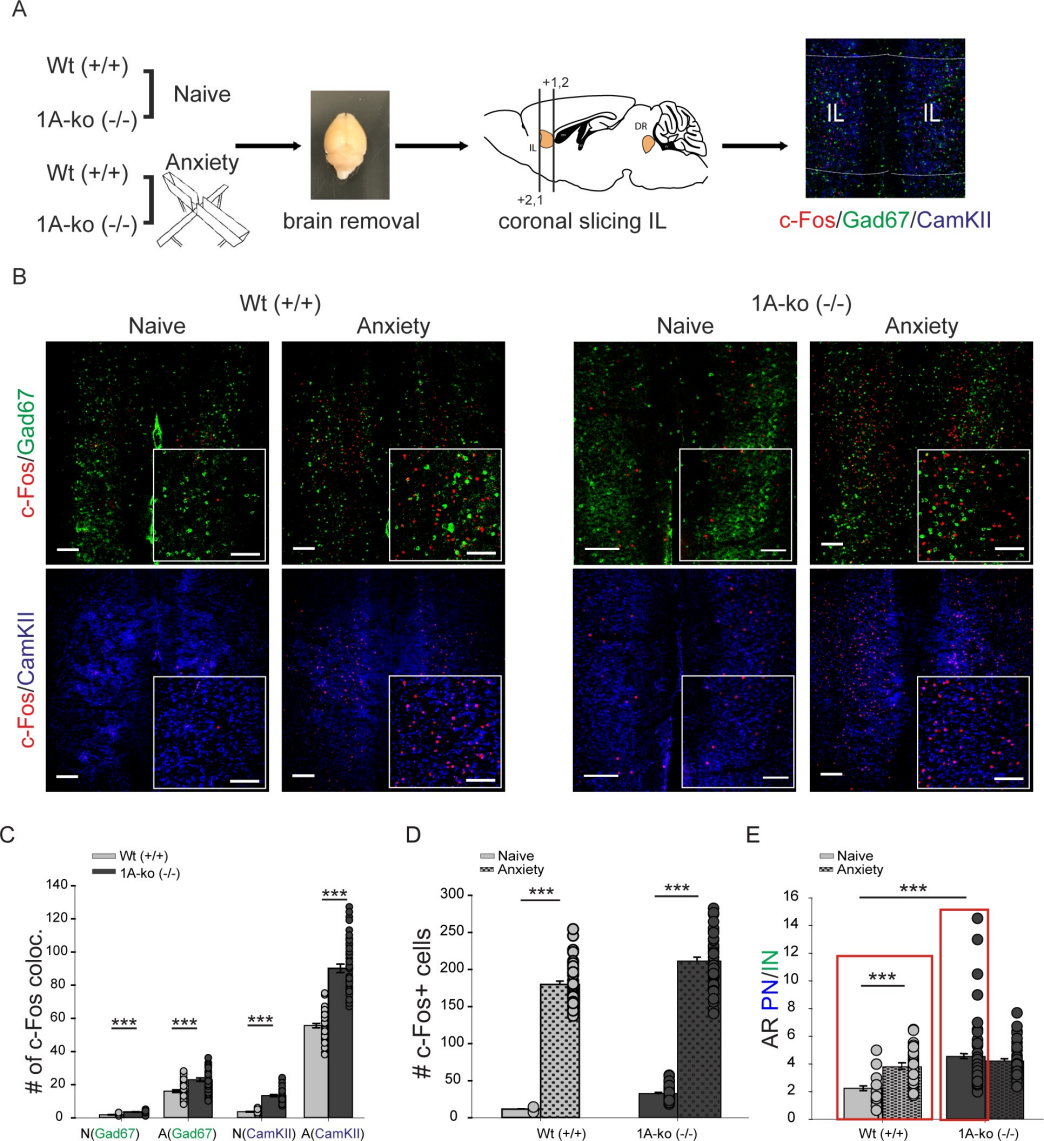
Elevation of cortical excitation during anxiety behavior. **(A)** experimental workflow of c-fos stainings. IL infralimbic cortex, Gad67 fluorescence labeling of GABAergic neurons (green), CamKII fluorescence labeling of pyramidal neurons (blue). **(B)** confocal images showing c-fos expression in Wt and 1A-ko. C-fos in red. Insets show high magnification. Scale bar 150μm, insets 100μm. **(C)** colocalization of c-fos expression in GABAergic and pyramidal cells (analysis of 42 brain slices in 3 brains per group). N(GAD67) Wt 1.82±0.13, 1A-ko 3.52±0.2, Mann Whitney Rank Sum test, p≤0.001, A(GAD67) Wt 16.1±0.84, 1A-ko 23.92±1.07, Mann Whitney Rank Sum test, p≤0.001, N(CamKII) Wt 3.68±0.2, 1A-ko 13.4±0.61, Mann Whitney Rank Sum test, p≤0.001, A(CamKII) Wt 55.64±1.29, 1A-ko 90.19±2.55, Mann Whitney Rank Sum test, p≤0.001. **(D)** total amount of c-fos expressing cells. Wt naïve 11.64±0.45, 1A-ko naïve 32.74±1.45, Mann Whitney Rank Sum test, p≤0.001, Wt anxiety 179.9±4.6, 1A-ko anxiety 211.33±5.36, Mann Whitney Rank Sum test, p≤0.001. **(E)** activity ratio between pyramidal neurons and interneurons (AR PN/IN), calculated as ratio between c-fos positive pyramidal and c-fos positive GABAergic neurons. Wt naïve 2.24±0.17, Wt anxiety 3.8±0.18, Mann Whitney Rank Sum p≤0.001, 1A-ko naïve 4.53±0.42,1A-ko anxiety 4.2±0.18 Mann Whitney Rank Sum p = 0.291, Wt naïve:1A-ko naïve, Mann Whitney Rank Sum test p≤0.001. Values are mean ± S.E.M. *** indicate significant differences (p≤ 0.001). Red boxes show significant difference between Wt and 1A-ko.

To test now whether anxiety may originate in the prefrontal cortex we examined c-fos expression in the IL (Fig 2). Again we found a highly significant increase of total c-fos in fearful situation in Wt, as well as in 5-HT_1A_(-/-) mice (Wt naïve 11.64±0.45, 1A-ko naïve 32.74±1.45, p≤0.001, Wt anxiety 179.9±4.6, 1A-ko anxiety 211.33±5.36, p≤0.001.) (Fig 2A–2D). The number of c-fos positive cells is increased in pyramidal (CamkII) and GABAergic interneurons (Fig 2C and 2D). But now a significant change in the ratio between active glutamatergic pyramidal neurons and active interneurons (AR PN/IN) between naïve conditions and anxiety is evident in Wt mice (Fig 2E). In Wt mice the AR PN/IN is nearly doubled (Wt naïve 2.24±0.17, Wt anxiety 3.8± 0.18, p≤0.001) during anxiety (Fig 2E), suggesting that a certain balance between excitation and inhibition, in the IL is important in the regulation of anxiety behaviour. Consequently, in 5-HT_1A_(-/-) knockout mice this AR PN/IN values are already elevated in naïve conditions and do not further increase in anxiety (1A-ko naïve 5.53±0.42 1A-ko anxiety 4.2±0.18, p = 0.291)(Fig 2E).This would suggest that probably enhanced anxiety levels in 1A-ko and Wt mice is triggered by a change in cortical E/I balance.

### C-fos expression in the amygdala during anxiety

**Fig 3:**
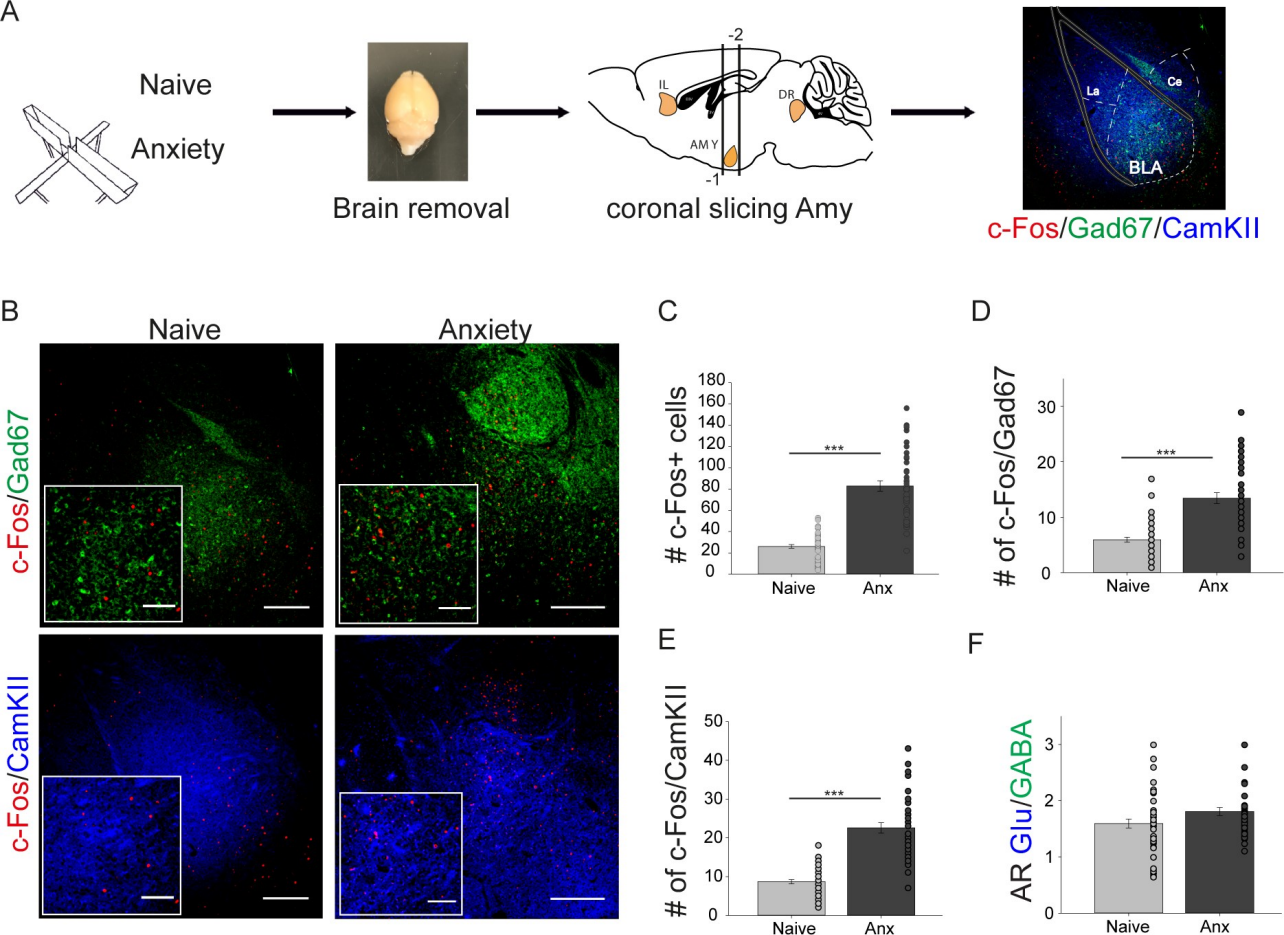
AR Glu/GABA in the basolateral amygdala is not changed due to anxiety. **(A)** experimental workflow of c-fos stainings in the amygdala. AMY amygdala, BLA basolaterale amygdala, Ce central amygdala, DR dorsal raphe, IL infralimbic cortex, La lateral amygdala. Gad67 fluorescence labeling of GABAergic neurons (green), CamKII fluorescence labeling of pyramidal neurons (blue), c-fos fluorescence labeling of c-fos expressing cells (red). **(B)** confocal images showing c-fos expression in the amygdala of naïve and anxiety treated mice. Gad67 fluorescence labeling of GABAergic neurons (green), CamKII fluorescence labeling of pyramidal neurons (blue), c-fos fluorescence labeling of c-fos expressing cells (red). Scale bar 250μm, Insets high magnification, scale bar 100μm. **(C)** total amount of c-fos expressing cells (analysis of 56 brain slices in 4 brains for naïve conditions and 42 brain slices in 3 brains for the anxiety condition): naïve 26.07±1.69, Anx 82.9±4.87, Mann Whitney Rank Sum test p≤0.001. **(D)** colocalization of c-fos expression in GABAergic cells: Naïve 5.95±0.44, Anx 13.45±0.98, Mann Whitney Rank Sum test p≤0.001. **(E)** colocalization of c-fos expression in pyramidal cells: naïve 8.72±0.55, Anx 22.57±1.34, Mann Whitney Rank Sum test p≤0.001. **(F)** activity ratio between glutamatergic neurons and interneurons (AR Glu/GABA), calculated as ratio between c-fos positive pyramidal and c-fos positive GABAergic neurons: naïve 1.59±0.08, Anx 1.81±0.07, Mann Whitney Rank Sum test, p = 0.075 n.s. Values are mean ± SEM. *** indicate significant differences (p≤0.001).

As the serotonergic system and the infralimbic cortex are both interconnected with the amygdala (S3 Fig) we decided to analyse also c-fos expression with in the amygdala in naive and anxiety conditions. As expected we found a highly significant increase of total c-fos in fearful situation in mice (naïve 26.07±1.69, Wt anxiety 82.9±4,87, p≤0.001.) (Fig 3B–3E). The number of c-fos positive cells is increased in GABAergic interneurons as well as in CamKII positive neurons (Fig 3D and 3E). Overall ratios between c-fos expressing glutamatergic and c-fos expressing GABAergic neurons (AR Glu/GABA) are not changed in the amygdala due to anxiety (Wt naïve 1.59±0.083, Wt anxiety 1.81±0.07, p = 0.078) (Fig 3F).

### Optogenetic excitation of pyramidal neurons in the IL promotes anxiety

**Fig 4:**
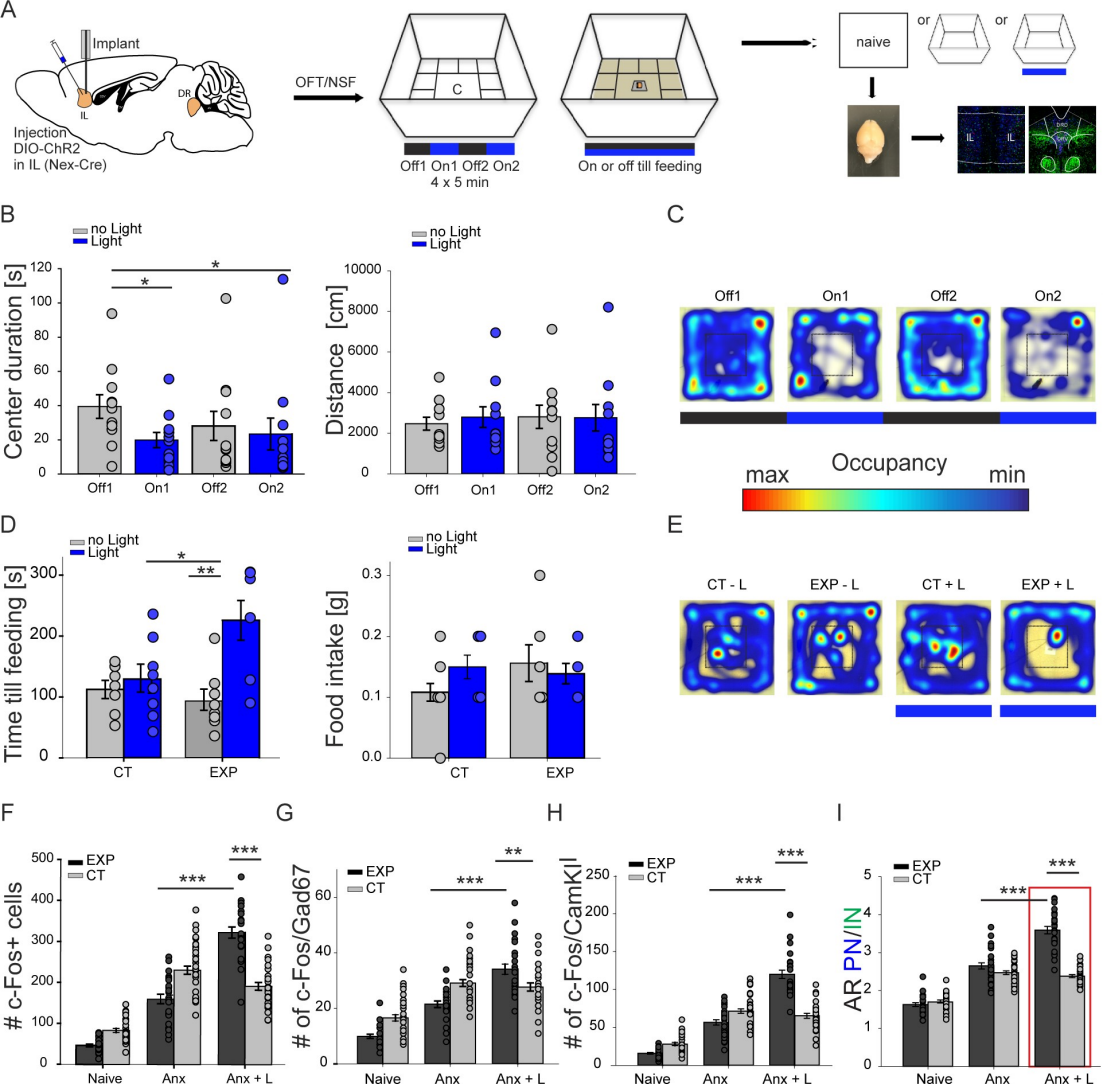
Elevated cortical AR PN/IN balance drives anxiety behavior. **(A)** targeting ChR2 to pyramidal cortical neurons and experimental workflow. IL infralimbic cortex **(B)** group data for openfield experiments in ChR2 injected animals (EXP), center duration Off1 39.49±6.9s, On1 19.87±4.47s, Off2 28.13±8.55s, On2 23.42±9.32s, Off1:On1 two tailed t-test, t = 2.286, p = 0.033, Off1:Off2 Mann Whitney Rank Sum test p = 0.168, Off1:On2 Mann Whitney Rank Sum test p = 0.049, distance moved Off1 2703.09±292.65cm, On1 3113.4±491.15cm, Off2 3331.86±482.62cm, On2 3082.17±658,61cm, Off1:On1 Mann Whitney Rank Sum test p = 0.743, Off1:Off2 two tailed t-test t = 0.52, p = 0.609, Off1:On2 Mann Whitney Rank Sum p = 0.646, n = 11 mice. **(C)** example of raw data from an individual mouse during an openfield test. **(D)** group data for novelty suppressed feeding, time till feeding CT no light 112.42±14.9s, CT light 129.38±22.96s, EXP no light 93.38±17.41s, EXP light 225.86±32.76s, CT light:EXP light, two tailed t-test, t = -2.461, p = 0.029, EXP no light:EXP light Mann Whitney Rank Sum test p = 0.004, food intake CT no light 0.12±0.02g, CT light 0.15±0.02g, EXP no light 0.16±0.03, EXP light 0.15±0.02 CT light:EXP light Mann Whitney Rank Sum test, p = 1, EXP no light:EXP light Mann Whitney Rank Sum test p = 0.955. **(E)** example of raw data from an individual mouse during novelty suppressed feeding test. **(F)** total amount of c-fos expressing cells, naïve EXP 47.11±3.25, CT 83.86±5, Anx EXP 160.14±10.14, CT 231.21±9.93, Anx+L EXP 323.38±13.61, CT 192.11±9.51. Anx EXP:Anx+L EXP two tailed-t-test t = -9.709, p≤0.001, Anx+L EXP:Anx+L CT two tailed-t-test t = -8, p≤0.001. Anx anxiety paradigm, Anx+L anxiety paradigm with light stimulation **(G)** colocalization of c-fos expression in GABAergic neurons (Gad67) naïve EXP 9.93±0.76, CT 16.57±1.17, Anx EXP 21.5±1.2, CT 29.14±1.23, Anx+L EXP 34.12±1.84, CT 27.71±1.49. Anx EXP:Anx+L EXP Mann Whitney Rank Sum test, p≤0.001, Anx+L EXP:Anx+L CT two tailed-t-test t = -2.714, p = 0.009. **(H)** colocalization of c-fos expression in pyramidal neurons (CamKII) naïve EXP 16.04±1.14, CT 28.21±2.1, Anx EXP 56.68±3.52, CT 71.57±2.98, Anx+L EXP 120.08±542, CT 65.46±3.26. Anx EXP:Anx+L EXP Mann Whitney Rank Sum test, p≤0.001, Anx+L EXP:Anx+L CT Mann Whitney Rank Sum test p≤0.001. **(I)** activity ratio between pyramidal neurons and interneurons (AR PN/IN), calculated as ratio between c-fos positive pyramidal and c-fos positive GABAergic neurons. Naïve EXP 1.63±0.05, CT 1.7±0.04, Anx EXP 2.65±0.08, CT 2.47±0.05, Anx+L EXP 3.59±0.1 CT 2.38±0.04. Anx EXP: Anx+L EXP two tailed t-test t = -7.377, p≤0.001, Anx+L EXP: Anx+L CT Mann Whitney Rank Sum test p≤0.001.Values are mean ± S.E.M. * indicate significant differences (p≤ 0.05), ** indicate significant differences (p≤ 0.01), *** indicate significant differences (p≤ 0.001).

To directly probe the role of increased cortical excitation, i.e. elevation of AR PN/IN in the IL, we optogenetically excited specifically pyramidal neurons during the OFT and the NSF and analysed c-fos immunolabeling subsequently (Fig 4A). We expressed ChR2 in pyramidal neurons of the left IL (S4 Fig) and implanted an optical fiber directly above the IL to specifically increase pyramidal neuron activity and therefore increase the AR PN/IN of the IL region (S4 Fig). In the OFT we alternated light stimulation with control phases in which no light stimulation occurred (Fig 4B and 4C). Stimulation of pyramidal neurons resulted in decreased center times (Off1 39.49±6.9s, On1 19.87±4.47s, Off2 28.13±8.55s, On2 23.42±9.32s) in comparison to the first control phase (Fig 4B). During the second control phase anxiety behaviour did not recover completely, but showed only a non-significant increase in center duration times. Control mice, in which dt-tomato was only expressed in pyramidal neurons, showed no alteration in anxiety behaviour due to light stimulation (S5A Fig). Light stimulation had no effects on motor behaviour (S4B and S5A Figs) and did not induce long lasting changes in anxiety behaviour (S5B Fig). Next we scrutinized the influence of excitation of IL pyramidal neurons in the NSF. Now we stimulated pyramidal neurons permanently during the execution of the NSF. After 24 h of food deprivation animals were challenged with a food pellet situated in the center of an OFT. Control animals, expression of dt-tomato with and without light stimulation, as well as ChR2 injected mice without light stimulation showed significant shorter latencies to start feeding than ChR2 injected mice with light stimulation (CT no light 112.42±14.9s, CT light 129.38±22.96s, EXP no light 93.38±17.41s, EXP light 225.86±32.76s) (Fig 4D and 4E) as also higher center duration times (S5C Fig). While excitation of pyramidal neurons increased latencies to feed more than twofold (EXP no light 93.38±17.41s, EXP light 225.86±32.76s) (Fig 4D) it divided center time duration by four (S5C Fig). Overall food intake was not changed among groups (CT no light 0.12±0.02g, CT light 0.15±0.02g, EXP no light 0.16±0.03, EXP light 0.15±0.02 CT) (Fig 4D). In addition we also conducted the EPM in ChR2 injected and control mice (S6 Fig). But unfortunately we noticed that during the execution of the EPM 42% of experimental and control animals fall off the maze. These animals had to be excluded from our analyses. All other animals, which did not fall off immediately had difficulties to walk on the open arms: they regularly slipped off from the open arms and then started consequently to avoid them categorically. Due to this behaviour control animals and experimental animals started to avoid open arms during all consecutive trials (S6A–S6C Fig). As this observed behaviour was completely independent from stimulation protocols (light or no light) or from the injected virus (ChR2 or control fluorophore) (S6D–S6F Fig), we think decreased open arm times in experimental, as well as in control mice do not permit to make any statement on the anxiety behaviour in the EPM. Taken together our results demonstrate that excitation of IL pyramidal neurons during the OFT and NSF enhance anxiety (Fig 4B–4E).

### C-Fos expression indicates that an elevated AR PN/IN in the IL induces anxiety

Subsequent immunohistochemical analyses in the IL revealed again increased c-fos expression in glutamatergic and GABAergic neurons in fearful situation (Fig 4F–4I), with even higher neuronal activity in glutamatergic neurons during additional light stimulation (naïve EXP 47.11±3.25, Anx EXP 160.14±10.14, Anx+L EXP 323.38±13.6). In control animals light stimulation had no further effects (Anx CT 231.21±9.93, ANX+L CT 192.11±9.51) (Fig 4F–4I). Consequently AR PN/IN is increased during light stimulation in experimental mice (Anx EXP 2.65±0.08, Anx+L EXP 3.59±0.1) and did not further change in control mice (AnxCT 2.47±0.05, Anx+L CT 2.38±0.04) (Fig 4I). Hence shifting the activity ratio between excitatory pyramidal neurons and inhibitory interneurons in the IL towards excitation promotes anxiety behaviour. To ensure, that optogenetic stimulation within the IL does not induce any stimulation artefacts within the DRN, which probably promote anxiety behaviour we additionally analysed AR 5-HT/GABA in the DRN during optogenetic stimulation in the IL. Light stimulation in the IL does not induce any significant changes in the activity ratio of the DRN (S7 Fig), emphasizing the pivotal role of IL cortex in the modulation of anxiety levels.

## Discussion

By combining behavioural and immunohistochemistry studies we could show, that in a model of anxiety an imbalance of excitatory pyramidal neurons and inhibitiory interneuron activity in the IL is evident and that optogenetic elevation of the pyramidal neuron activity in the IL is sufficient to resemble this phenotype and drives anxiety behaviour. In conjunction with current models of anxiety our study provides further evidence that a functional decrease of serotonergic neurotransmission at postsynaptic sites and subsequent disinhibition of IL pyramidal neurons may be a pathogenic mechanism of anxiety [9,26].

### Circuitry of anxiety

Previous studies have identified the prefrontal cortex as part of a tripartite anxiety network, consisting of the amygdala, the ventral hippocampus (vHPC) and the prefrontal cortex. Manipulations within this network are able to chance anxiety behaviour [4,20–22]. During the expression of anxiety, activity between the vHPC and the prefrontal cortex are highly synchronized and power in theta frequencies (4–12 Hz) is markedly increased in an anxiogenic environment [22,35]. Adhikari et al. (2011) hypothesize that place fields within the vHPC are well suited to encode the emotional valence of the environment and can provide contextual information to the mPFC [35], which in turn can act on the amygdala to modulate exploratory behaviour. In general the PFC is associated with a role in active inhibition of behaviour (Shah and Treit 2003) and it is an intriguing hypothesize that the PFC is by this connectivity able to modulate the expression of anxiety behaviour. Single unit recordings in mPFC have revealed that neurons in the mPFC encode safe and aversive places in the EPM [36], pointing again to the involvement of PFC neurons in guiding exploratory behaviour.

Our results are in conjunction with studies that report large increases in theta power and prefrontal cortex firing rates in 5-HT_1A_(-/-) knockout and high avoidance Wt mice during anxiety [35,37]. Presumably, in our experiments an acute optogenetic increase in pyramidal neuronal activity amplified theta synchrony, which in turn leads then to an enhanced expression of anxiety by the generalization of aversiveness throughout the anxiety probing test. This might also explain, that in contrast to a study by Fuchikami et al. 2015, in which a continuous light stimulation of layer 5 pyramidal neurons over 60 minutes one day prior to testing induced anxiolytic and antidepressant behavioural responses [38], acute light stimulation of pyramidal neurons in all cortical layers during a stressful situation is anxiogenic. Acute changes in pyramidal neuron activity might directly act on anxiety behaviour by modulating the encoding of safe vs. aversive places, whereas spine sprouting in the prefrontal cortex by ketamine or optogenetic stimulation could probably prime the prefrontal cortex to less inhibition of exploratory behaviour.

### The role of the prefrontal cortex in anxiety

Contradictory results have been obtained regarding the role of the PFC in anxiety. Studies report a decrease in anxiety after lesioning of the PFC [6,39], but also anxiogenic behaviour was observed after inhibition of the PFC [15,16]. In the course of this conflicting results differential roles of subdivisions of the PFC, i.e. the IL and prelimbic cortex (PL) were proposed [40].

Pharmacological manipulations of inhibitory and excitatory neurotransmission within the PFC indicated that enhanced excitatory inputs to the PFC, i.e. increased glutamate or decreased GABA neurotransmission and therewith an imbalance of excitation inhibition, support anxiety behaviour [9]. These results are in tight conjunction with our results and underlie the notion that disturbances in cortical excitation inhibition balance promote pathological conditions and are one possible cause of anxiety disorders [41–44]. Two recent studies have revealed the importance of projections and connections to the IL in extinction learning [7] pointing to the complex role of the PFC in anxiety and fear regulation and underlining probably different mechanism in innate anxiety and acquired anxiety.

### Serotonin and anxiety

Numerous studies have investigated the influence of serotonergic neurotransmission on anxiety. One of the most famous genetic mouse models of anxiety is a 5-HT_1A(-/-)_ receptor knockout mouse line. This mouse line provides further evidence for a close link of the serotonergic system with anxiety. Pharmacogenetic studies revealed that an increase in serotonergic neuronal activity results in elevated anxiety (Teissier et al. 2015) whereas depletion of 5-HT reduces anxiety [18,45]. As serotonin preferentially acts on 5-HT_1A_ receptors of GABAergic interneurons, the net effect of serotonin release into the cortex results in inhibition of GABAergic interneurons and therefore in a disinhibition of pyramidal neurons, which is associated with an increase in pyramidal neuron activity [46]. Hence serotonin would be a potential candidate to initiate a transition from balanced excitation/inhibition ratios to enhanced excitation. Our observations of an enhanced AR PN/IN in 5-HT_1A(-/-)_ mice is in accordance with former studies, which reported an important role of 5-HT_1A_ receptors in tuning E/I balance [47].

However, pre- and postsynaptic effects of serotonin in the prefrontal cortex might act differently on anxiety. A study by Kjaerby et al. 2016 convincingly showed that presynaptic 5-HT_1B_ receptors are able to selectively supress hippocampal and callosal input leading to an inhibition of prefrontal theta oscillations and in turn to reduced anxiety mediated avoidance [48]. It is likely that disturbed serotonergic neurotransmission could cause changes in the balance between excitatory and inhibitory neuronal activity ratios by altering synaptic input strength or by incorrect gating of inputs.

Postsynaptically mainly 5-HT_1A_ receptors, located on pyramidal, as well as on GABAergic interneurons, are expressed in the prefrontal cortex. Several studies, in humans and animal models have shown that, especially dysfunction of postsynaptic 5-HT_1A_ receptors is associated with anxiety [49–52]. Albert et al. 2014 propose an anxiety model, in which either too low or too high levels of serotonin will induce anxiety by an increased pyramidal neuron output, mediated by the highly sensitive 5-HT_1A_ receptor [18]. Our studies support this anxiety model and underlines further the necessity of balanced E/I in the prefrontal cortex for healthy anxiety behaviour.

### Conclusion

Our findings suggest that an exact balance between excitatory pyramidal neurons and inhibitory interneurons in the IL of the prefrontal cortex is a prerequisite to maintain healthy anxiety responses and that an increased activity of cortical pyramidal neurons can drive anxiety. Follow-up studies have to proof if shifting this ratio towards interneuron activity will already be anxiolytic or if specifically, 5-HT_1A_ signalling in the IL is needed to maintain normal anxiety levels. Nevertheless an oversimplification should be avoided, i.e. excitation and inhibition might be regulated differently at different circuits levels, for example neurons in different microcircuits targeting other brain areas could be modulated independently and excitatory and inhibitory inputs might even vary at different cell compartments [53].

Our study further supports that a dysregulation of AR PN/IN in cortical areas, presumably caused by disturbed serotonergic signalling, is one possible cause of generalized anxiety giving new insights into underlying mechanism of anxiety.

## Supporting information

S1 Fig Enhanced anxiety in 5-HT_1A(-/-)_ knockout mice. **(A)** setup Elevated-Plus Maze (EPM) **(B)** group data for the EPM, time in open arms: Wt 47.91±6.12s, 1A-ko 31.59±4.34s, two-tailed t-test, t = 2.207, p = 0.026, Wt n = 10, 1A-ko n = 18 **(C)** group data for the EPM open arm entries: 10.6±1.25, 1A-ko 7.28±0.66, two-tailed t-test, t = 2.604, p = 0.015, n = 10, 1A-ko n = 18. **(D)** group data for distance moved Wt 3859.34±979.26cm, 1A-ko 4373±669.08cm, Mann Whitney Rank Sum test, p = 0.487, Wt n = 8, 1A-ko n = 18. **(E)** setup novelty suppressed feeding (NSF). **(F)** group data for NSF time in center Wt = 27.32±3.81s, 1A-ko = 17.57±2.6s, two-tailed t-test, t = 2.193, p = 0.042, Wt n = 8, 1A-ko = 12. **(G)** group data NSF time till feeding Wt 118.56±19.16s, 1A-ko = 146.69±36.67s, Mann Whitney Rank Sum test, p = 0.772, Wt n = 10, 1A-ko = 16. **(H)** NSF food intake, Wt 0.1±0.015g, 1A-ko 0.13±0.027g, Mann Whitney Rank Sum test, p = 0.613. Values are mean ± S.E.M. * indicate significant differences (p≤ 0.05).
(PDF)

S2 Fig Connectivity of the IL and DRN. **(A)** experimental workflow for retrograde tracing of DRN-IL projection neurons and confocal images showing fluorogold stained cell bodies in the DRN. Only the left IL was injected. Fluorogold stained cell bodies in the DRN (yellow) and immunohistochemical labeling of TPH positive neurons (blue). Scale bar 1mm. Insets show high magnification. Scale bar 50μm. Arrow heads indicate exemplarily double-positive cells. **(B)** experimental workflow for anterograde tracing of IL-DRN projections and confocal image showing expression of td-tomato in fibers terminating in the DRN. TPH stained cell bodies in the DRN (blue) and fiber terminals (red). Scale bar 500μm. Insets show high magnification. Gad67 stained cell bodies in the DRN (green) and fiber terminals (red). Scale bar 50μm. **(C)** experimental workflow for retrograde tracing of IL-DRN projection neurons and confocal image showing fluorogold stained cells bodies (yellow) in the IL. Only the left IL was in injected. Fluorogold stained cell bodies in the IL (yellow) and immunohistochemical labeling of CamKII positive neurons (blue). Scale bar 1mm. Insets show high magnification. Scale bar 50μm. Arrows indicate double-positive cells. DRD dorsal raphe drsal part, DRV dorsal raphe ventral part, IL infralimbic cortex.
(PDF)

S3 Fig Connectivity of the amygdala with the IL and the DRN. **(A)** experimental workflow for anterograde tracing of the left IL-amygdala projections and confocal image showing expression of td-tomato fibers from the IL terminating in the left amygdala (top) or the right amygdala (bottom). CamKII stained cell bodies in the amygdala (blue). Gad67 stained cell bodies in the amygdala (green) and fiber terminals from the IL (red). Scale bar 250μm. Insets of high magnification show either the colocalization of CamKII or Gad67 with fiber terminals arising from the IL. Scale bar 100 μm. **(B)** experimental workflow for retrograde tracing of IL-amygdala projection neurons and confocal images showing fluorogold stained cell bodies (yellow), flurogold was injected in the left amygdala. Fluorogold stained cell bodies in the IL (yellow) and immunohistochemical labeling of CamKII positive neurons (blue). Scale bar 500μm. Insets show colocalization of fluorogold and CamKII in high magnification, scale bar 100μm. **(C)** experimental workflow for retrograde tracing of DRN-amygdala projection neurons and confocal images showing fluorogold stained cell bodies (yellow), fluorogold was injected in the left amygdala. Fluorogold stained cell bodies in the IL (yellow) and immunohistochemical labeling of TPH positive neurons (blue). Scale bar 500μm. Insets show colocalization of fluorogold and TPH in high magnification, scale bar 100μm. Arrows indicate examples for double positive cells. AMY amygdala, DR dorsal raphe, DRD dorsal raphe dorsal part, DRV dorsal raphe central part, IL infralimbic cortex,
(PDF)

S4 Fig Injections sites and expression in pyramidal neurons. **(A)** ChR2 or td-tomato was unilateral delivered into the left IL, optical fibers were placed in the IL and optical stimulation with blue (473 nm) light was restricted to the IL. **(B)** Confocal images showing expression of ChR2 (left, yellow) and td-tomato (right, red) in the IL. Cell nuclei are marked with DAPI (blue). Scale bar 1mm. **(C)** Insets from b showing high magnification of the IL region. Scale bar 150μm. Cell nuclei are marked with DAPI (blue) **(D)** Top row, Colocalization of ChR2 (yellow) with pyramidal neurons (CamKII in blue). Scale bar 150μm. Bottom row, high magnification of above confocal images showing in detail the injected and non-injected side. Scale bar 100μm. Arrow heads indicate exemplarily double-positive cells. **(E)** Top row, ChR2 (yellow) is not expressed in GABAergic neurons (Gad67 in green). Scale bar 150μm. Bottom row, high magnification of above confocal images showing in detail the injected and non-injected side. Scale bar 100μm. IL infralimbic cortex, PL prelimbic cortex.
(PDF)

S5 Fig Control experiments. **(A)** group data for the OFT in control mice. Center duration Off1 16.73±2.65s, On1 16.02±1.89s, Off2 12.02±1.76s, On2 13.04±2.58s, Mann Whitney Rank Sum Off1:On1 p = 0.868, Off1:Off2 p = 0.263, Off1:On2 p = 0.33. Distance moved Off1 3399.69±296.77cm, On1 3210.6±446.9cm, Off2 3030.28±513.83cm, On2 2955.82±617.7, Mann Whitney Rank Sum test Off1:On1 p = 0.171, Off1:Off2 p = 0.081, Off1:On2 p = 0.028, n = 15. **(B)** group data for the NSF. Center duration CT no light 5.8±2.01s, CT light 8.85±3.11s, EXP no light 7.93±2.79s, EXP light 1.55±0.81s. CT no light:CT light two tailed t-test, t = -0.844, p = 0.417, EXP no light:EXP light Mann Whitney Rank Sum test p = 0.035, CT light:EXP light Whitney Rank Sum p = 0.053. CT no light n = 6, CT light n = 7, EXP no light n = 6, EXP light n = 7. **(C)** group data for persistent effects of anxiety in CT and EXP mice. Two days after the OFT mice were again challenged in the openfield. Neither freezing, nor center times were significantly different in CT or EXP mice, indicating that optogenetic stimulation did not have long-term effects on anxiety behavior. Freezing: CT 3.36±1.16s, EXP 5.4±2, two tailed t-test t = -0.957, p = 0.364, Center duration CT 6.89±0.89s, EXP 6.17±1.38s, two tailed t-test t = 0,461, p = 0.656, CT n = 7, EXP n = 4. Values are mean ± S.E.M. * indicate significant differences (p≤ 0.05).
(PDF)

S6 Fig Nex-Cre mice failed to perform Elevated-Plus maze test. **(A)** setup Elevated-Plus Maze and light stimulation protocol. **(B)** group data for “slip off’s” (slipping off with back paws from the floor of the open arm) for experimental and control animals. Only mice, which stayed all trials in the EPM are considered. Slip offs in first light off phase are reason for later avoidance of open arms in all groups: Off1 EXP 1.625±0.6, CT 2.2±0.79, Off2+3 EXP 0.125±0.125, CT 0±0, On1+2+3 EXP 0.625±0.26, CT 0.1±0.1. Pie chart of mice falling from the EPM 42,42% fall down and only 57,57% were able to stay in the EPM during all trials. **(C)** group data for the experimental group in the EPM, time in open arms: Off1 73.91±12.22s, On1 36.15±14.65s, Off2 15.61±6.23s, On2 19.49±7.51s, Off3 9.36±4.44s, On3 7.96±3.47s, n = 12, two-tailed t-test Off1:On1 p = 0,041, t = 2.168. **(E)** group data for the control group in the EPM, time in open arms: Off1 86.92±12.74s, On1 33.78±14.38s, Off2 18.01±11.61s, On2 16.41±9.61, Off3 11.36±4.01, On3 5.43±2.07, n = 11, Mann Whitney Rank Sum test Off1:On1 p = 0.009. **(F)** group data for distance moved by experimental animals, no significant differences were evident during trials: Off1 679.96±71.63cm, On1 712.24±112.82cm, Off2 717.49±97.39cm, On2 782.51±81.11cm, Off3 722.11±68.60cm, On3 663.90±106.57cm, n = 12. **(G)** group data for distance moved by control animals, no significant differences were evident during trials: Off1 705.11±88.36cm, On1 789.45±77.53cm, Off2 724.74±80.49cm, On2 676.57±111.99cm, Off3 716.99±132.47cm, On3 663.03±132.46cm, n = 11. Values are mean ± SEM. * indicate significant differences (p = ≤0.05), ** indicate significant differences (p≤0.01). CT control animals, EXP experimental animals.
(PDF)

S7 Fig E/I balance in the serotonergic system is not changed due to optogenetic activation of cortical pyramidal cells. **(A)** total amount of c-fos expressing cells in the DRN: naïve EXP 49.43±2.91, CT 48.29±3.28, Anx EXP 81.93±3.68, CT 77.21±3.26, Anx+L EXP 73.88±4.33, CT 79.54±5.12, two-tailed t-test EXP naïve: EXP Anx p≤0.001, t = -6.928, n = 28, two-tailed t-test CT naïve:CT Anx p≤0.001, t = -6.253, n = 28. **(B)** colocalization of c-fos expression in GABAergic neurons (Gad67): naïve EXP 5.64±0.4, CT 5.79±0.49, Anx EXP 8.71±0.54, CT 8.11±0.5, Anx+L EXP 7.78±0.51, CT 8.0±0.66, two-tailed t-test EXP naïve:EXP Anx p≤0.001, t = 4.539, n = 28, two-tailed t-test CT naïve:CT Anx p = 0.002, t = -3.286, n = 28. **(C)** colocalization of c-fos expression in pyramidal neurons (CamKII): naïve EXP 11.32±18.21, CT 11.21±0.82, Anx EXP 20.18±1.04, CT 17.61±0,78, Anx+L EXP 18.21±0.92, CT 17.39±1.26, Mann Whitney Rank Sum test EXP naïve:EXP Anx p≤0.001, n = 28, two-tailed t-test CT naïve:CT Anx p≤0.001, t = -5.663, n = 28. **(D)** Excitation/Inhibition balance, calculated as ratio between c-fos positive serotonergic neurons as main projection neurons and c-fos positive GABAergic neurons, no significant difference is evident between conditions: naïve EXP 2.16±0.11, CT 2.04±0.09, Anx EXP2.44±0.12, CT 2.32±0.13, Anx+L EXP 2.52±0.16, CT 2.28±0.09. Values are mean ± SEM. ** indicate significant differences (p = ≤0.01), *** indicate significant differences (p≤0.001). Anx anxiety condition, Anx+L anxiety condition with light stimulation, CT control animals, EXP experimental mice injected with ChR2.
(PDF)
