# Supplementary material for: Enhanced activity of pyramidal neurons in the infralimbic cortex drives anxiety behavior
Source: PLoS One. 2019 Jan 24;14(1):e0210949. doi: 10.1371/journal.pone.0210949 (PMC6345483; doi:10.1371/journal.pone.0210949)
Supplement: S1 Fig — (A) setup Elevated-Plus Maze (EPM) (B) group data for the EPM, time in open arms: Wt 47.91±6.12s, 1A-ko 31.59±4.34s, two-tailed t-test, t = 2.207, p = 0.026, Wt n = 10, 1A-ko n = 18 (C) group data for the EPM open arm entries: 10.6±1.25, 1A-ko 7.28±0.66, two-tailed t-test, t = 2.604, p = 0.015, n = 10, 1A-ko n = 18. (D) group data for distance moved Wt 3859.34±979.26cm, 1A-ko 4373±669.08cm, Mann Whitney Rank Sum test, p = 0.487, Wt n = 8, 1A-ko n = 18. (E) setup novelty suppressed feeding (NSF). (F) group data for NSF time in center Wt = 27.32±3.81s, 1A-ko = 17.57±2.6s, two-tailed t-test, t = 2.193, p = 0.042, Wt n = 8, 1A-ko = 12. (G) group data NSF time till feeding Wt 118.56±19.16s, 1A-ko = 146.69±36.67s, Mann Whitney Rank Sum test, p = 0.772, Wt n = 10, 1A-ko = 16. (H) NSF food intake, Wt 0.1±0.015g, 1A-ko 0.13±0.027g, Mann Whitney Rank Sum test, p = 0.613. Values are mean ± S.E.M. * indicate significant differences (p≤ 0.05). (PDF) [file pone.0210949.s001.pdf]

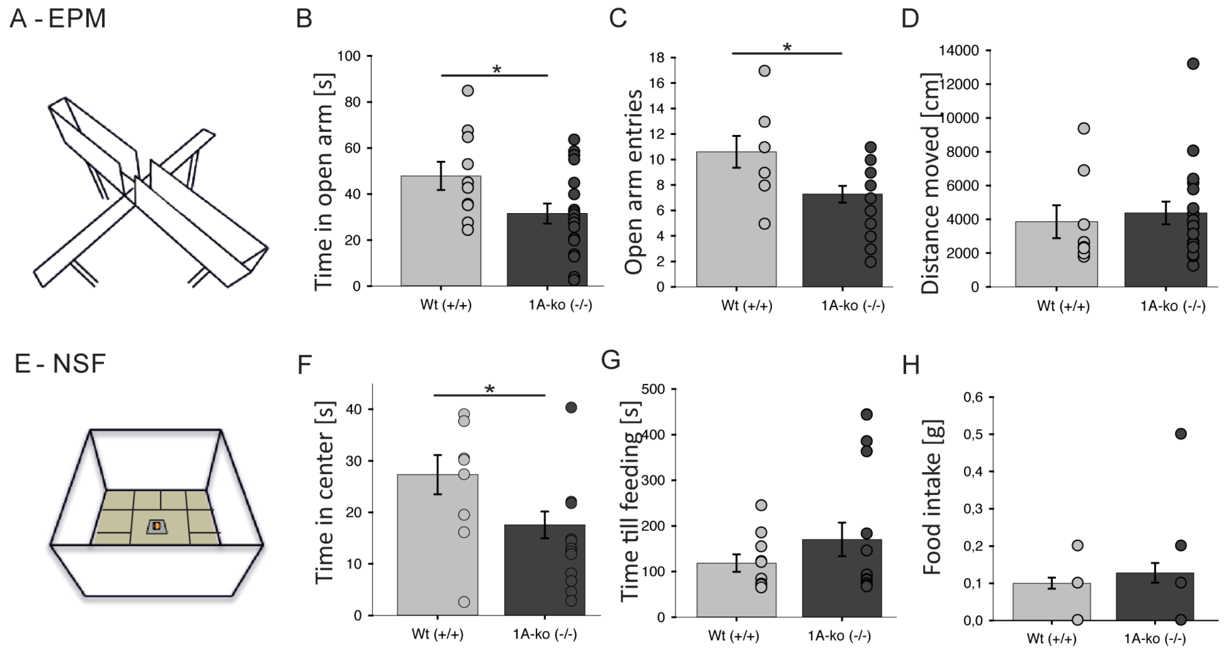

S1 Fig. Enhanced anxiety in 5-$HT_{1A(-/-)}$ knockout mice

(A) setup Elevated-Plus Maze (EPM) **(B)** group data for the EPM, time in open arms: Wt 47.91±6.12s, 1A-ko 31.59±4.34s, two-tailed t-test, t=2.207, p=0.026, Wt n=10, 1A-ko n=18 **(C)** group data for the EPM open arm entries: 10.6±1.25, 1A-ko 7.28±0.66, two-tailed t-test, t=2.604, p=0.015, n=10, 1A-ko n=18. **(D)** group data for distance moved Wt 3859.34±979.26cm, 1A-ko 4373±669.08cm, Mann Whitney Rank Sum test, p=0.487, Wt n=8, 1A-ko n=18. **(E)** setup novelty suppressed feeding (NSF). **(F)** group data for NSF time in center Wt=27.32±3.81s, 1A-ko=17.57±2.6s, two-tailed t-test, t=2.193, p=0.042, Wt n=8, 1A-ko=12. **(G)** group data NSF time till feeding Wt 118.56±19.16s, 1A-ko=146.69±36.67s, Mann Whitney Rank Sum test, p=0.772, Wt n=10, 1A-ko=16. **(H)** NSF food intake, Wt 0.1±0.015g, 1A-ko 0.13±0.027g, Mann Whitney Rank Sum test, p=0.613. Values are mean ± S.E.M. * indicate significant differences (p≤ 0.05).
