# Supplementary material for: Enhanced activity of pyramidal neurons in the infralimbic cortex drives anxiety behavior
Source: PLoS One. 2019 Jan 24;14(1):e0210949. doi: 10.1371/journal.pone.0210949 (PMC6345483; doi:10.1371/journal.pone.0210949)
Supplement: S2 Fig — (A) experimental workflow for retrograde tracing of DRN-IL projection neurons and confocal images showing fluorogold stained cell bodies in the DRN. Only the left IL was injected. Fluorogold stained cell bodies in the DRN (yellow) and immunohistochemical labeling of TPH positive neurons (blue). Scale bar 1mm. Insets show high magnification. Scale bar 50μm. Arrow heads indicate exemplarily double-positive cells. (B) experimental workflow for anterograde tracing of IL-DRN projections and confocal image showing expression of td-tomato in fibers terminating in the DRN. TPH stained cell bodies in the DRN (blue) and fiber terminals (red). Scale bar 500μm. Insets show high magnification. Gad67 stained cell bodies in the DRN (green) and fiber terminals (red). Scale bar 50μm. (C) experimental workflow for retrograde tracing of IL-DRN projection neurons and confocal image showing fluorogold stained cells bodies (yellow) in the IL. Only the left IL was in injected. Fluorogold stained cell bodies in the IL (yellow) and immunohistochemical labeling of CamKII positive neurons (blue). Scale bar 1mm. Insets show high magnification. Scale bar 50μm. Arrows indicate double-positive cells. DRD dorsal raphe drsal part, DRV dorsal raphe ventral part, IL infralimbic cortex. (PDF) [file pone.0210949.s002.pdf]

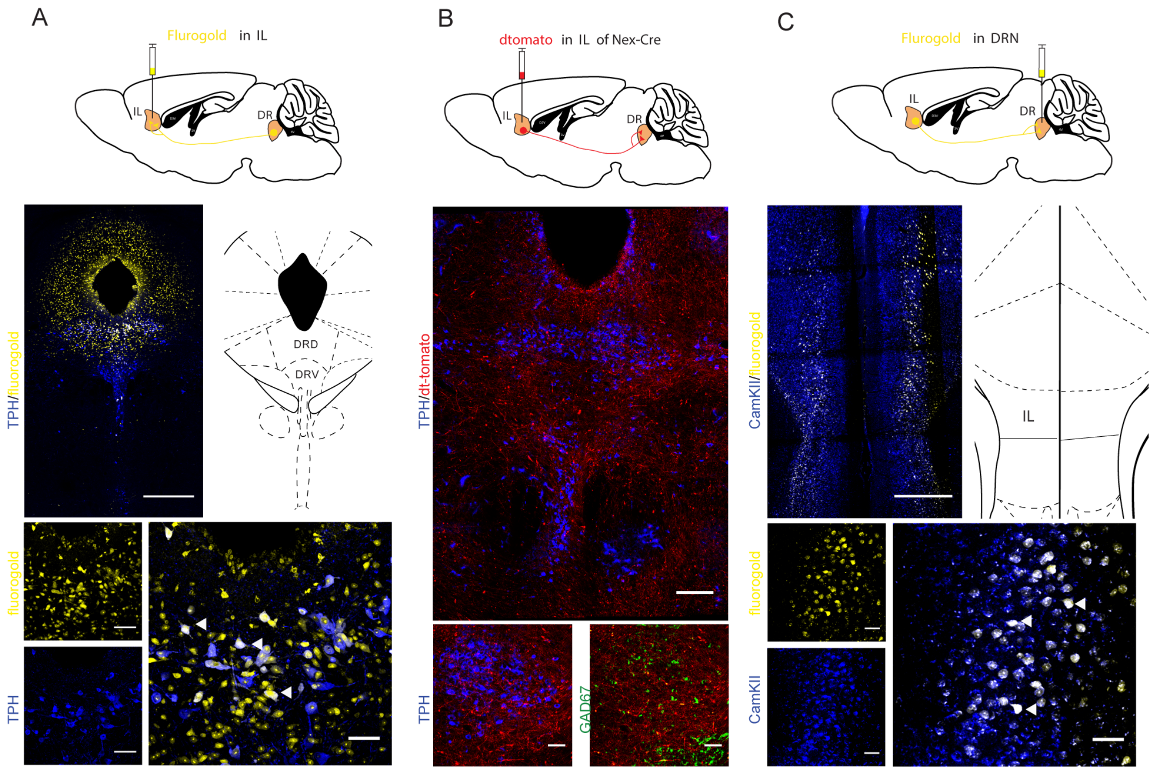

S2 Fig. Connectivity of the IL and DRN

(A) experimental workflow for retrograde tracing of DRN-IL projection neurons and confocal images showing fluorogold stained cell bodies in the DRN. Only the left IL was injected. Fluorogold stained cell bodies in the DRN (yellow) and immunohistochemical labeling of TPH positive neurons (blue). Scale bar 1mm. Insets show high magnification. Scale bar 50µm. Arrow heads indicate exemplarily double-positive cells. **(B)** experimental workflow for anterograde tracing of IL-DRN projections and confocal image showing expression of td-tomato in fibers terminating in the DRN. TPH stained cell bodies in the DRN (blue) and fiber terminals (red). Scale bar 500µm. Insets show high magnification. Gad67 stained cell bodies in the DRN (green) and fiber terminals (red). Scale bar 50µm. **(C)** experimental workflow for retrograde tracing of IL-DRN projection neurons and confocal image showing fluorogold stained cells bodies (yellow) in the IL. Only the left IL was in injected. Fluorogold stained cell bodies in the IL (yellow) and immunohistochemical labeling of CamKII positive neurons (blue). Scale bar 1mm. Insets show high magnification. Scale bar 50µm. Arrows indicate double-positive cells. DRD dorsal raphe drsal part, DRV dorsal raphe ventral part, IL infralimbic cortex.
