# Supplementary material for: Enhanced activity of pyramidal neurons in the infralimbic cortex drives anxiety behavior
Source: PLoS One. 2019 Jan 24;14(1):e0210949. doi: 10.1371/journal.pone.0210949 (PMC6345483; doi:10.1371/journal.pone.0210949)
Supplement: S3 Fig — (A) experimental workflow for anterograde tracing of the left IL-amygdala projections and confocal image showing expression of td-tomato fibers from the IL terminating in the left amygdala (top) or the right amygdala (bottom). CamKII stained cell bodies in the amygdala (blue). Gad67 stained cell bodies in the amygdala (green) and fiber terminals from the IL (red). Scale bar 250μm. Insets of high magnification show either the colocalization of CamKII or Gad67 with fiber terminals arising from the IL. Scale bar 100 μm. (B) experimental workflow for retrograde tracing of IL-amygdala projection neurons and confocal images showing fluorogold stained cell bodies (yellow), flurogold was injected in the left amygdala. Fluorogold stained cell bodies in the IL (yellow) and immunohistochemical labeling of CamKII positive neurons (blue). Scale bar 500μm. Insets show colocalization of fluorogold and CamKII in high magnification, scale bar 100μm. (C) experimental workflow for retrograde tracing of DRN-amygdala projection neurons and confocal images showing fluorogold stained cell bodies (yellow), fluorogold was injected in the left amygdala. Fluorogold stained cell bodies in the IL (yellow) and immunohistochemical labeling of TPH positive neurons (blue). Scale bar 500μm. Insets show colocalization of fluorogold and TPH in high magnification, scale bar 100μm. Arrows indicate examples for double positive cells. AMY amygdala, DR dorsal raphe, DRD dorsal raphe dorsal part, DRV dorsal raphe central part, IL infralimbic cortex, (PDF) [file pone.0210949.s003.pdf]

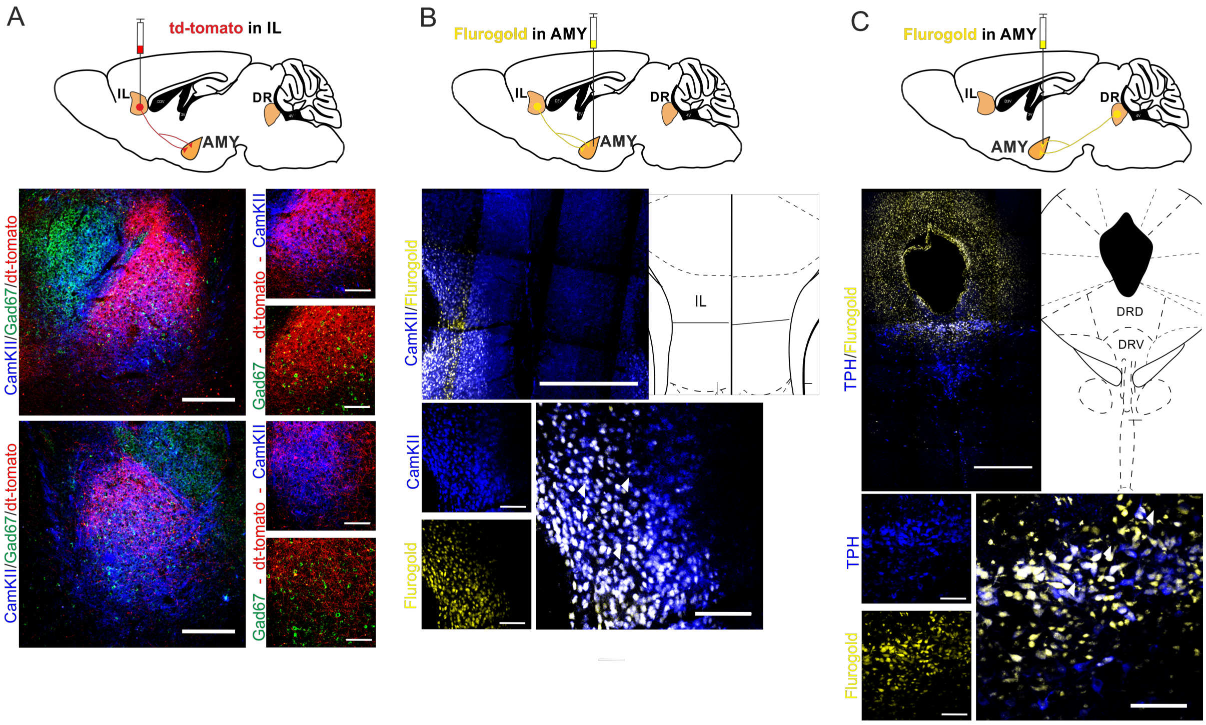

S3 Fig. Connectivity of the amygdala with the IL and the DRN

(A) experimental workflow for anterograde tracing of the left IL-amygdala projections and confocal image showing expression of td-tomato fibers from the IL terminating in the left amygdala (top) or the right amygdala (bottom). CamKII stained cell bodies in the amygdala (blue). Gad67 stained cell bodies in the amygdala (green) and fiber terminals from the IL (red). Scale bar 250µm. Insets of high magnification show either the colocalization of CamKII or Gad67 with fiber terminals arising from the IL. Scale bar 100 µm. **(B)** experimental workflow for retrograde tracing of IL-amygdala projection neurons and confocal images showing fluorogold stained cell bodies (yellow), flurogold was injected in the left amygdala. Fluorogold stained cell bodies in the IL (yellow) and immunohistochemical labeling of CamKII positive neurons (blue). Scale bar 500µm. Insets show colocalization of fluorogold and CamKII in high magnification, scale bar 100µm. **(C)** experimental workflow for retrograde tracing of DRN-amygdala projection neurons and confocal images showing fluorogold stained cell bodies (yellow), fluorogold was injected in the left amygdala. Fluorogold stained cell bodies in the IL (yellow) and immunohistochemical labeling of TPH positive neurons (blue). Scale bar 500µm. Insets show colocalization of fluorogold and TPH in high magnification, scale bar 100µm. Arrows indicate examples for double positive cells. AMY amygdala, DR dorsal raphe, DRD dorsal raphe dorsal part, DRV dorsal raphe central part, IL infralimbic cortex.
