# Supplementary material for: Enhanced activity of pyramidal neurons in the infralimbic cortex drives anxiety behavior
Source: PLoS One. 2019 Jan 24;14(1):e0210949. doi: 10.1371/journal.pone.0210949 (PMC6345483; doi:10.1371/journal.pone.0210949)
Supplement: S4 Fig — (A) ChR2 or td-tomato was unilateral delivered into the left IL, optical fibers were placed in the IL and optical stimulation with blue (473 nm) light was restricted to the IL. (B) Confocal images showing expression of ChR2 (left, yellow) and td-tomato (right, red) in the IL. Cell nuclei are marked with DAPI (blue). Scale bar 1mm. (C) Insets from b showing high magnification of the IL region. Scale bar 150μm. Cell nuclei are marked with DAPI (blue) (D) Top row, Colocalization of ChR2 (yellow) with pyramidal neurons (CamKII in blue). Scale bar 150μm. Bottom row, high magnification of above confocal images showing in detail the injected and non-injected side. Scale bar 100μm. Arrow heads indicate exemplarily double-positive cells. (E) Top row, ChR2 (yellow) is not expressed in GABAergic neurons (Gad67 in green). Scale bar 150μm. Bottom row, high magnification of above confocal images showing in detail the injected and non-injected side. Scale bar 100μm. IL infralimbic cortex, PL prelimbic cortex. (PDF) [file pone.0210949.s004.pdf]

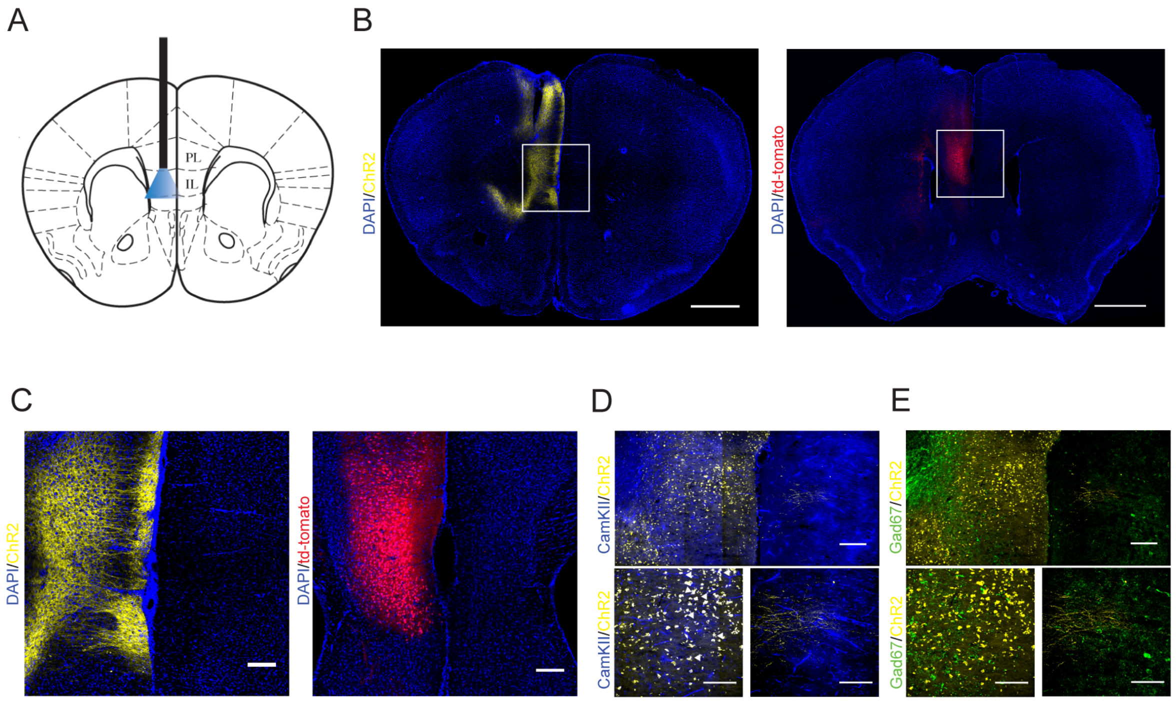

S4 Fig. Injections sites and expression in pyramidal neurons

(A) ChR2 or td-tomato was unilateral delivered into the left IL, optical fibers were placed in the IL and optical stimulation with blue (473 nm) light was restricted to the IL. **(B)** Confocal images showing expression of ChR2 (left, yellow) and td-tomato (right, red) in the IL. Cell nuclei are marked with DAPI (blue). Scale bar 1mm. **(C)** Insets from b showing high magnification of the IL region. Scale bar 150µm. Cell nuclei are marked with DAPI (blue) **(D)** Top row, Colocalization of ChR2 (yellow) with pyramidal neurons (CamKII in blue). Scale bar 150µm. Bottom row, high magnification of above confocal images showing in detail the injected and non-injected side. Scale bar 100µm. Arrow heads indicate exemplarily double-positive cells. **(E)** Top row, ChR2 (yellow) is not expressed in GABAergic neurons (Gad67 in green). Scale bar 150µm. Bottom row, high magnification of above confocal images showing in detail the injected and non-injected side. Scale bar 100µm. IL infralimbic cortex, PL prelimbic cortex.
