# Supplementary material for: Enhanced activity of pyramidal neurons in the infralimbic cortex drives anxiety behavior
Source: PLoS One. 2019 Jan 24;14(1):e0210949. doi: 10.1371/journal.pone.0210949 (PMC6345483; doi:10.1371/journal.pone.0210949)
Supplement: S5 Fig — (A) group data for the OFT in control mice. Center duration Off1 16.73±2.65s, On1 16.02±1.89s, Off2 12.02±1.76s, On2 13.04±2.58s, Mann Whitney Rank Sum Off1:On1 p = 0.868, Off1:Off2 p = 0.263, Off1:On2 p = 0.33. Distance moved Off1 3399.69±296.77cm, On1 3210.6±446.9cm, Off2 3030.28±513.83cm, On2 2955.82±617.7, Mann Whitney Rank Sum test Off1:On1 p = 0.171, Off1:Off2 p = 0.081, Off1:On2 p = 0.028, n = 15. (B) group data for the NSF. Center duration CT no light 5.8±2.01s, CT light 8.85±3.11s, EXP no light 7.93±2.79s, EXP light 1.55±0.81s. CT no light:CT light two tailed t-test, t = -0.844, p = 0.417, EXP no light:EXP light Mann Whitney Rank Sum test p = 0.035, CT light:EXP light Whitney Rank Sum p = 0.053. CT no light n = 6, CT light n = 7, EXP no light n = 6, EXP light n = 7. (C) group data for persistent effects of anxiety in CT and EXP mice. Two days after the OFT mice were again challenged in the openfield. Neither freezing, nor center times were significantly different in CT or EXP mice, indicating that optogenetic stimulation did not have long-term effects on anxiety behavior. Freezing: CT 3.36±1.16s, EXP 5.4±2, two tailed t-test t = -0.957, p = 0.364, Center duration CT 6.89±0.89s, EXP 6.17±1.38s, two tailed t-test t = 0,461, p = 0.656, CT n = 7, EXP n = 4. Values are mean ± S.E.M. * indicate significant differences (p≤ 0.05). (PDF) [file pone.0210949.s005.pdf]

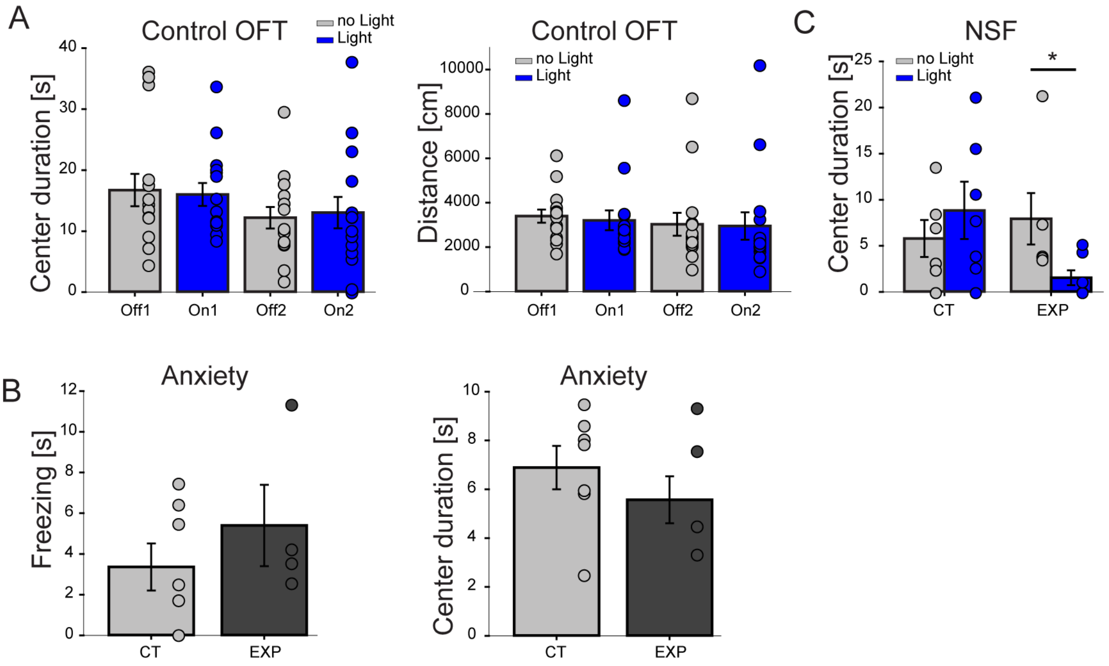

S5 Fig. Control experiments

(A) group data for the OFT in control mice. Center duration Off1 16.73±2.65s, On1 16.02±1.89s, Off2 12.02±1.76s, On2 13.04±2.58s, Mann Whitney Rank Sum Off1:On1 p=0.868, Off1:Off2 p=0.263, Off1:On2 p=0.33. Distance moved Off1 3399.69±296.77cm, On1 3210.6±446.9cm, Off2 3030.28±513.83cm, On2 2955.82±617.7, Mann Whitney Rank Sum test Off1:On1 p=0.171, Off1:Off2 p=0.081, Off1:On2 p=0.028, n=15. **(B)** group data for persistent effects of anxiety in CT and EXP mice. Two days after the OFT mice were again challenged in the openfield. Neither freezing, nor center times were significantly different in CT or EXP mice, indicating that optogenetic stimulation did not have long-term effects on anxiety behavior. Freezing: CT 3.36±1.16s, EXP 5.4±2, two tailed t-test t=-0.957, p=0.364, Center duration CT 6.89±0.89s, EXP 6.17±1.38s, two tailed t-test t=0,461, p=0.656, CT n=7, EXP n=4. Values are mean ± S.E.M. * indicate significant differences (p≤ 0.05). **(C)** group data for the NSF. Center duration CT no light 5.8±2.01s, CT light 8.85±3.11s, EXP no light 7.93±2.79s, EXP light 1.55±0.81s. CT no light:CT light two tailed t-test, t=-0.844, p=0.417, EXP no light:EXP light Mann Whitney Rank Sum test p=0.035, CT light:EXP light Whitney Rank Sum p=0.053. CT no light n=6, CT light n=7, EXP no light n=6, EXP light n=7.
