# Supplementary material for: Enhanced activity of pyramidal neurons in the infralimbic cortex drives anxiety behavior
Source: PLoS One. 2019 Jan 24;14(1):e0210949. doi: 10.1371/journal.pone.0210949 (PMC6345483; doi:10.1371/journal.pone.0210949)
Supplement: S6 Fig — (A) setup Elevated-Plus Maze and light stimulation protocol. (B) group data for “slip off’s” (slipping off with back paws from the floor of the open arm) for experimental and control animals. Only mice, which stayed all trials in the EPM are considered. Slip offs in first light off phase are reason for later avoidance of open arms in all groups: Off1 EXP 1.625±0.6, CT 2.2±0.79, Off2+3 EXP 0.125±0.125, CT 0±0, On1+2+3 EXP 0.625±0.26, CT 0.1±0.1. Pie chart of mice falling from the EPM 42,42% fall down and only 57,57% were able to stay in the EPM during all trials. (C) group data for the experimental group in the EPM, time in open arms: Off1 73.91±12.22s, On1 36.15±14.65s, Off2 15.61±6.23s, On2 19.49±7.51s, Off3 9.36±4.44s, On3 7.96±3.47s, n = 12, two-tailed t-test Off1:On1 p = 0,041, t = 2.168. (E) group data for the control group in the EPM, time in open arms: Off1 86.92±12.74s, On1 33.78±14.38s, Off2 18.01±11.61s, On2 16.41±9.61, Off3 11.36±4.01, On3 5.43±2.07, n = 11, Mann Whitney Rank Sum test Off1:On1 p = 0.009. (F) group data for distance moved by experimental animals, no significant differences were evident during trials: Off1 679.96±71.63cm, On1 712.24±112.82cm, Off2 717.49±97.39cm, On2 782.51±81.11cm, Off3 722.11±68.60cm, On3 663.90±106.57cm, n = 12. (G) group data for distance moved by control animals, no significant differences were evident during trials: Off1 705.11±88.36cm, On1 789.45±77.53cm, Off2 724.74±80.49cm, On2 676.57±111.99cm, Off3 716.99±132.47cm, On3 663.03±132.46cm, n = 11. Values are mean ± SEM. * indicate significant differences (p = ≤0.05), ** indicate significant differences (p≤0.01). CT control animals, EXP experimental animals. (PDF) [file pone.0210949.s006.pdf]

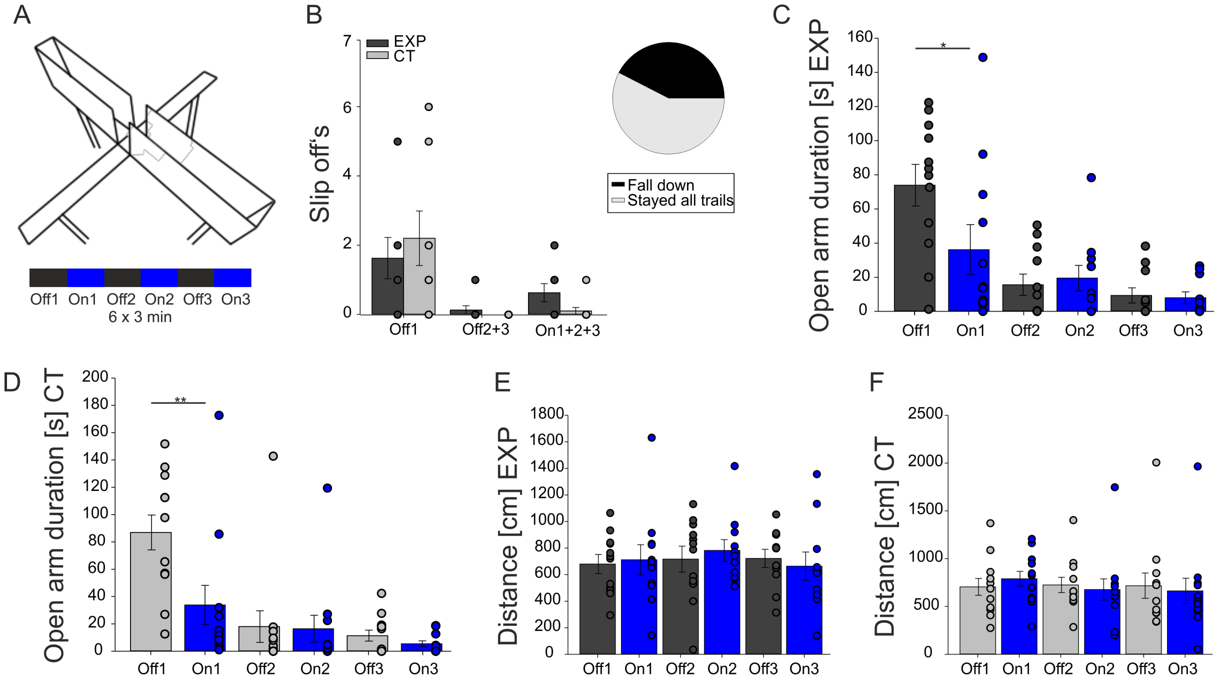

S6 Fig. Nex-Cre mice failed to perform Elevated-Plus Maze Test

(A) setup Elevated-Plus Maze and light stimulation protocol. **(B)** group data for "slip off's" (slipping off with back paws from the floor of the open arm) for experimental and control animals. Only mice, which stayed all trials in the EPM are considered. Slip offs in first light off phase are reason for later avoidance of open arms in all groups: Off1 EXP 1.625±0.6, CT 2.2±0.79, Off2+3 EXP 0.125±0.125, CT 0±0, On1+2+3 EXP 0.625±0.26, CT 0.1±0.1. Pie chart of mice falling from the EPM 42,42% fall down and only 57,57% were able to stay in the EPM during all trials. **(C)** group data for the experimental group in the EPM, time in open arms: Off1 73.91±12.22s, On1 36.15±14.65s, Off2 15.61±6.23s, On2 19.49±7.51s, Off3 9.36±4.44s, On3 7.96±3.47s, n=12, two-tailed t-test Off1:On1 p=0,041, t=2.168. **(D)** group data for the control group in the EPM, time in open arms: Off1 86.92±12.74s, On1 33.78±14.38s, Off2 18.01±11.61s, On2 16.41±9.61, Off3 11.36±4.01, On3 5.43±2.07, n=11, Mann Whitney Rank Sum test Off1:On1 p=0.009. **(E)** group data for distance moved by experimental animals, no significant differences were evident during trials: Off1 679.96±71.63cm, On1 712.24±112.82cm, Off2 717.49±97.39cm, On2 782.51±81.11cm, Off3 722.11±68.60cm, On3 663.90±106.57cm, n=12. **(F)** group data for distance moved by control animals, no significant differences were evident during trials: Off1 705.11±88.36cm, On1 789.45±77.53cm, Off2 724.74±80.49cm, On2 676.57±111.99cm, Off3 716.99±132.47cm, On3 663.03±132.46cm, n=11. Values are mean ± SEM. * indicate significant differences (p=≤0.05), ** indicate significant differences (p≤0.01). CT control animals, EXP experimental animals.
