# Supplementary material for: Enhanced activity of pyramidal neurons in the infralimbic cortex drives anxiety behavior
Source: PLoS One. 2019 Jan 24;14(1):e0210949. doi: 10.1371/journal.pone.0210949 (PMC6345483; doi:10.1371/journal.pone.0210949)
Supplement: S7 Fig — (A) total amount of c-fos expressing cells in the DRN: naïve EXP 49.43±2.91, CT 48.29±3.28, Anx EXP 81.93±3.68, CT 77.21±3.26, Anx+L EXP 73.88±4.33, CT 79.54±5.12, two-tailed t-test EXP naïve: EXP Anx p≤0.001, t = -6.928, n = 28, two-tailed t-test CT naïve:CT Anx p≤0.001, t = -6.253, n = 28. (B) colocalization of c-fos expression in GABAergic neurons (Gad67): naïve EXP 5.64±0.4, CT 5.79±0.49, Anx EXP 8.71±0.54, CT 8.11±0.5, Anx+L EXP 7.78±0.51, CT 8.0±0.66, two-tailed t-test EXP naïve:EXP Anx p≤0.001, t = 4.539, n = 28, two-tailed t-test CT naïve:CT Anx p = 0.002, t = -3.286, n = 28. (C) colocalization of c-fos expression in pyramidal neurons (CamKII): naïve EXP 11.32±18.21, CT 11.21±0.82, Anx EXP 20.18±1.04, CT 17.61±0,78, Anx+L EXP 18.21±0.92, CT 17.39±1.26, Mann Whitney Rank Sum test EXP naïve:EXP Anx p≤0.001, n = 28, two-tailed t-test CT naïve:CT Anx p≤0.001, t = -5.663, n = 28. (D) Excitation/Inhibition balance, calculated as ratio between c-fos positive serotonergic neurons as main projection neurons and c-fos positive GABAergic neurons, no significant difference is evident between conditions: naïve EXP 2.16±0.11, CT 2.04±0.09, Anx EXP2.44±0.12, CT 2.32±0.13, Anx+L EXP 2.52±0.16, CT 2.28±0.09. Values are mean ± SEM. ** indicate significant differences (p = ≤0.01), *** indicate significant differences (p≤0.001). Anx anxiety condition, Anx+L anxiety condition with light stimulation, CT control animals, EXP experimental mice injected with ChR2. (PDF) [file pone.0210949.s007.pdf]

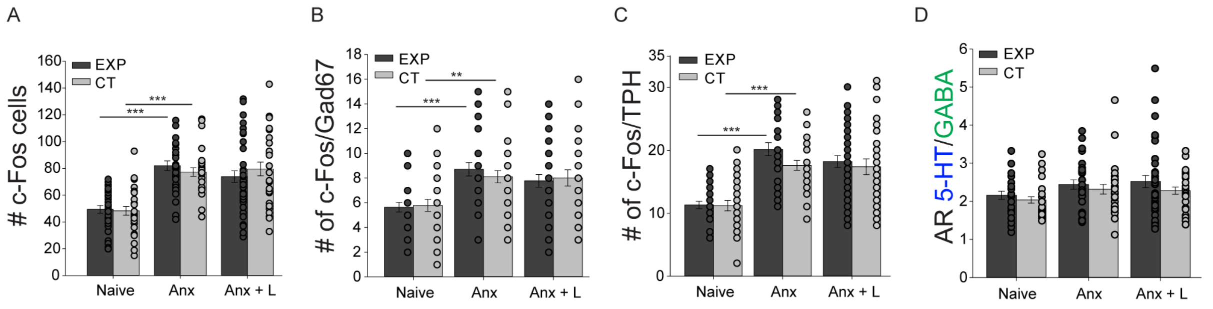

S7 Fig. AR 5-HT/GABA in the serotonergic system is not changed due to optogenetic activation of cortical pyramidal cells.

(A) total amount of c-fos expressing cells in the DRN: naïve EXP 49.43±2.91, CT 48.29±3.28, Anx EXP 81.93±3.68, CT 77.21±3.26, Anx+L EXP 73.88±4.33, CT 79.54±5.12, two-tailed t-test EXP naïve: EXP Anx p≤0.001, t=-6.928, n=28, two-tailed t-test CT naïve:CT Anx p≤0.001, t=-6.253, n=28. **(B)** colocalization of c-fos expression in GABAergic neurons (Gad67): naïve EXP 5.64±0.4, CT 5.79±0.49, Anx EXP 8.71±0.54, CT 8.11±0.5, Anx+L EXP 7.78±0.51, CT 8.0±0.66, two-tailed t-test EXP naïve:EXP Anx p≤0.001, t=4.539, n=28, two-tailed t-test CT naïve:CT Anx p=0.002, t=-3.286, n=28. **(C)** colocalization of c-fos expression in pyramidal neurons (CamKII): naïve EXP 11.32±18.21, CT 11.21±0.82, Anx EXP 20.18±1.04, CT 17.61±0,78, Anx+L EXP 18.21±0.92, CT 17.39±1.26, Mann Whitney Rank Sum test EXP naïve:EXP Anx p≤0.001, n=28, two-tailed t-test CT naïve:CT Anx p≤0.001, t=-5.663, n=28. **(D)** activity ration between serotonergic and interneurons, calculated as ratio between c-fos positive serotonergic neurons as main projection neurons and c-fos positive GABAergic neurons, no significant difference is evident between conditions: naïve EXP 2.16±0.11, CT 2.04±0.09, Anx EXP2.44±0.12, CT 2.32±0.13, Anx+L EXP 2.52±0.16, CT 2.28±0.09. Values are mean ± SEM. ** indicate significant differences (p=≤0.01), *** indicate significant differences (p≤0.001). Anx anxiety condition, Anx+L anxiety condition with light stimulation, CT control animals, EXP experimental mice injected with ChR2.
